# Tumor-Associated Macrophages Induce Endocrine Therapy Resistance in ER+ Breast Cancer Cells

**DOI:** 10.3390/cancers11020189

**Published:** 2019-02-06

**Authors:** Andrés M. Castellaro, María C. Rodriguez-Baili, Cecilia E. Di Tada, Germán A. Gil

**Affiliations:** 1 Departamento de Química Biológica, Facultad de Ciencias Químicas, CIQUIBIC CONICET, Universidad Nacional de Córdoba, Ciudad Universitaria, Córdoba X5000HUA, Argentina; acastellaro@fcq.unc.edu.ar (A.M.C.); mrodriguez@fcq.unc.edu.ar (M.C.R.-B.); 2 Laboratorio de Inmunohistoquímica, Fundación para el Progreso de la Medicina, Córdoba X5000EMS, Argentina; ceciliaditada@fpmlab.org.ar

**Keywords:** macrophages, tumor microenvironment, breast cancer, estrogen receptor, tamoxifen, endocrine resistance, TNF-α, IL-6, NF-κB

## Abstract

Antiestrogenic adjuvant treatments are first-line therapies in patients with breast cancer positive for estrogen receptor (ER+). Improvement of their treatment strategies is needed because most patients eventually acquire endocrine resistance and many others are initially refractory to anti-estrogen treatments. The tumor microenvironment plays essential roles in cancer development and progress; however, the molecular mechanisms underlying such effects remain poorly understood. Breast cancer cell lines co-cultured with TNF-α-conditioned macrophages were used as pro-inflammatory tumor microenvironment models. Proliferation, migration, and colony formation assays were performed to evaluate tamoxifen and ICI 182,780 resistance and confirmed in a mouse-xenograft model. Molecular mechanisms were investigated using cytokine antibody arrays, WB, ELISA, ChIP, siRNA, and qPCR-assays. In our simulated pro-inflammatory tumor microenvironment, tumor-associated macrophages promoted proliferation, migration, invasiveness, and breast tumor growth of ER+ cells, rendering these estrogen-dependent breast cancer cells resistant to estrogen withdrawal and tamoxifen or ICI 182,780 treatment. Crosstalk between breast cancer cells and conditioned macrophages induced sustained release of pro-inflammatory cytokines from both cell types, activation of NF-κB/STAT3/ERK in the cancer cells and hyperphosphorylation of ERα, which resulted constitutively active. Our simulated tumor microenvironment strongly altered endocrine and inflammatory signaling pathways in breast cancer cells, leading to endocrine resistance in these cells.

## 1. Introduction

Anti-estrogen adjuvant treatments are the first line of therapy for management of breast cancer in patients (>67% of total patients) whose tumors are estrogen receptor positive (ER+); such treatments reduce the degree of 15-year mortality by ~33% [1]. This therapy is based on use of various drugs that block estradiol receptors [2]. Proliferative effects of estradiol in breast tissue are modulated by members of the nuclear estrogen receptor (ER) family of transcription factors [3]. There are two ER subtypes (ERα and ERβ); ERα is the primary determinant of breast epithelial cell development and proliferation [4,5,6,7]. Selective ER modulators (SERMs) bind to intracellular ERs and act as agonists or antagonists, depending on the target organ. Tamoxifen, for example, is a pioneer SERM that blocks ER in breast tissue and has been widely used for treatment of ER+ breast cancers in pre- and post-menopausal women [8]. ICI 182,780 (a.k.a. Faslodex or Fulvestrant) is a pure antagonist that blocks ER activity in all tissues tested, prevents recruitment of transcriptional coactivators, and promotes interactions with corepressors [9,10,11]. Another frequently used strategy, blocking of estradiol production by aromatase inhibitors, may improve clinical efficacy in post-menopausal women [12,13]. Endocrine therapy is highly effective overall in patients with ER+ breast cancer; however, some patients display de novo resistance to the therapy, while others show initial benefit but eventually relapse with acquired endocrine resistance [14,15,16]. VC Jordan’s group found that only 17–28% of patients with acquired resistance to tamoxifen lost ERα expression [17].

Increasing attention in studies related to "endocrine resistance" (i.e., resistance to endocrine therapy) has been paid to interaction of ER with signaling pathways involving growth factor receptors such as epidermal growth factor receptor, ErbB2, insulin-like growth factor-1 receptor, and fibroblast growth factor receptor. Inhibition of such signaling pathways has successfully overcome endocrine resistance in many cases [18,19,20,21,22,23,24]. Other mechanisms of endocrine resistance include altered phosphorylation and activation of ERα [25,26,27,28], altered levels of transcriptional coregulators [29,30], upregulation of signaling factors such as NF-κB [31,32], c-Myc, and cyclin D1 [33,34], and enhancement of IL-6/STAT3 and other signaling pathways [35]. Growth factor signaling may result in deregulated expression of cell cycle stimulating genes (cyclin D, cyclin E, MYC) [36]. ERα regulates transcription of its targets through ligand-dependent recruitment of transcriptional coregulator proteins, including various histone-modifying enzymes [37,38,39,40]. Overexpression of NCOR1, a corepressor of ER, is associated with enhanced responsiveness to tamoxifen [41].

Few studies have addressed effects of the tumor microenvironment on induction of endocrine resistance. Bidirectional signaling between a tumor and the surrounding stroma plays a crucial role in tumor progression [42,43]. Tumor-associated macrophages (TAMs) typically comprise a high proportion of immune cells in tumors, and are associated with poor prognosis in breast cancer [44,45,46] because they promote tumor cell growth, tissue remodeling, angiogenesis, and suppression of immune responses [47,48,49,50]. Macrophages modulate various signaling pathways that tend to promote breast cancer progression, including pathways involved in production of growth factors, proinflammatory cytokines, and chemokines in the tumor microenvironment [51,52,53,54].

TNF-α is a pleiotropic cytokine produced by many different cell types, but is synthesized primarily by monocytic lineage cells such as macrophages [55]. TNF-α is a strong proinflammatory agent that regulates many facets of macrophage function, and is considered a “master regulator” of proinflammatory cytokine production and inflammatory cell activation, through the NF-κB and ERK pathways [56]. Macrophages facilitate hormone resistance of certain tumors (e.g., prostate) through IL-1β-induced NF-κB signaling [57], but the relevance of this pathway in breast cancer is unclear. The NF-κB oncoprotein family regulates transcription of genes involved in many aspects of tumor progression, including processes of angiogenesis, tissue remodeling, survival, and inflammatory response [58]. Notable transcriptional targets in the latter category are the inflammatory cytokines IL-6, IL-8, and CCL5 (RANTES) [59,60].

Estradiol suppresses NF-κB-responsive genes in breast cancer cells [61]. Effects of estradiol treatment on NF-κB signaling may be related to improved prognosis observed in some ER+ breast cancer patients. ER- breast cancer has been correlated with increased NF-κB activity [62,63] and increased expression of certain cytokines (IL-6, IL-8) and chemokines (CCL5, MCP-1 [CCL2]) [64,65,66].

Results presented here demonstrate that NF-κB- and IL-6-dependent signaling pathways play essential roles in macrophage-mediated induction of endocrine resistance in ER+ breast cancer cells, and the consequent ability of these cells to proliferate in estradiol-independent manner. These findings facilitate evaluation of joint ER/NF-κB signaling in breast tumors, and of the roles of macrophages in tumorigenesis.

## 2. Results

### 2.1. Macrophages Promote Proliferation, Invasion, and Migration of ER+ Breast Cancer Cells in an Estradiol-Independent Manner

**Figure 1:**
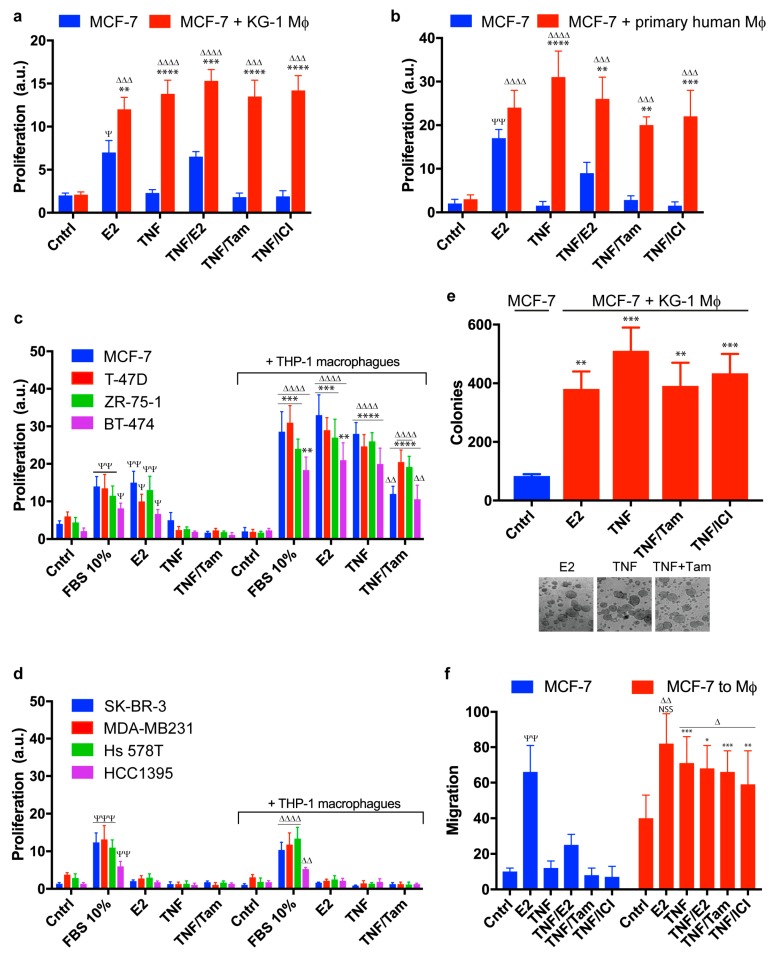
Macrophage-mediated endocrine resistance in breast cancer cells. (**a**,**b**) Proliferation of MCF-7 cells cultured in presence or absence of conditioned KG-1 macrophages (**a**) or primary human macrophages (**b**), measured by CyQUANT and indicated as arbitrary units (a.u). Cell cultures were separated by a semipermeable membrane (see Methods) and cultured for 2 days with the indicated ligands. *, in comparison with absence of macrophages in indicated treatment; Δ or Ψ, in comparison with respective Cntrl of each group. (**c**,**d**) Proliferation of ER+ (**c**) and ER− (**d**) breast cancer cell lines cultured in presence or absence of conditioned THP-1 macrophages, measured by CyQUANT. Notations as in panels (**a**,**b**). (**e**) Soft-agar colony formation assays of MCF-7 co-cultured with KG-1. Data shown are number of colonies formed after 21 days. Bottom: representative bright field micrograph. Ligands were added in fresh medium every 2 days for 3 weeks. Analysis in comparison with Cntrl. (**f**) Migration assays. MCF-7 were labeled with fluorophore and placed in the top of a transwell insert (pore size 8 µm), with or without unlabeled conditioned KG-1 macrophages in the bottom well. Fluorescence was measured in the bottom well after 48 h and expressed as a percentage. Fluorescence intensity equal to that from plating 1 × 10^5^ fluorescent cells directly in the lower well was defined as 100% migration, *n* = 3. Notations as in panels (**a**,**b**). Cntrl: Fresh DMEM, E2: Estradiol 1 nM, TNF: TNF-α 1 ng/mL, Tam: Tamoxifen 1 µM, ICI: ICI 182,780 1 µM. * *p* < 0.05; ** *p* < 0.01; *** *p* < 0. 001; **** *p* < 0. 0001; Δ *p* < 0.05; ΔΔ *p* < 0.01; ΔΔΔ *p* < 0.001; ΔΔΔΔ *p* < 0.0001; Ψ *p* < 0.05; ΨΨ *p* < 0.01; ΨΨΨ *p* < 0.001; NSS: not statistically significant.

To elucidate the effects of macrophages on endocrine-responsive breast cancer cells, we co-cultured MCF-7 cells with macrophages, using a semipermeable membrane (pore size 0.4 µm) to separate the two cell lines. This membrane prevents passage of cells, but allows passage of cytokines and other solutes. Primary human macrophages or KG-1 macrophages pretreated with TNF-α (conditioned macrophages) were used as described in Methods. Proliferation rate of MCF-7 cultured alone was increased by estradiol (E2) and reduced by tamoxifen or ICI 182,780 treatment, as expected. TNF-α-treated MCF-7 did not proliferate (Figure 1a,b, blue bars; Figure S1a). In contrast, TNF-α-treated MCF-7 co-cultured with conditioned KG-1 or primary human macrophages did proliferate, even in the absence of estradiol or the presence of tamoxifen or ICI 182,780. In the presence of added estradiol, proliferation rate of MCF-7 co-cultured with conditioned KG-1 or THP-1 macrophages was greater than that of MCF-7 cultured alone (Figure 1a, red bars; Figure S1b), and such increase for cells co-cultured with conditioned primary human macrophages was present but not statistically significant (Figure 1b). Such modulation was also observed for ER+ breast cell lines other than MCF-7 (Figure 1c and Figure S1c), indicating that this effect of conditioned macrophages is not cell type-specific. The modulation was evidently pendent, since ER- breast cell lines treated with TNF-α and co-cultured with conditioned THP-1 macrophages did not proliferate (Figure 1d). The fact that the modulation was clearly observed for KG-1 macrophages, THP-1 macrophages, and primary human macrophages (Figure 1a–c and Figure S1b,c) demonstrates that conditioned macrophages from various sources can promote breast cancer endocrine resistance. Conditioning of macrophages with TNF-α was necessary in order to induce notable proliferation of ER+ breast cancer cells in the absence of estradiol or presence of antagonists; i.e., nonconditioned THP-1 macrophages had no such effect (Figure S1d). Under our experimental conditions using conditioned macrophages, proliferation was induced in several ER+ cell lines, but not in ER- cell lines. M. Detmar’s group reported seemingly contradictory results [67]; however, their study involved different co-culture conditions, testing of only two cell lines (T47D, MDA-MB-231), and direct contact between primary monocytes and tumor cells.

TNF-α is a strong proinflammatory agent involved in regulation of many aspects of macrophage function and proinflammatory cytokine production. Our observations that ER+ breast cancer cells grew in the absence of estradiol, and even in the presence of ER antagonists when co-cultured with conditioned macrophages, suggested that macrophages may mediate endocrine resistance. To clarify the role of macrophages in tumorigenesis of these cancer cells, we examined invasiveness and migration in vitro. MCF-7 cells alone cultured in soft agar formed few colonies (<5 per well), whereas MCF-7 co-cultured with conditioned KG-1 macrophages displayed strikingly increased colony formation that was not inhibited by tamoxifen or ICI 182,780 (Figure 1e).

Similar results were obtained in migration experiments. MCF-7 migration was assessed using a transwell insert with semipermeable membrane (pore size 8 µm). Pre-stained cells with fluorophore were placed in the upper well, and fluorescence of cells that reached the lower well by passing through the membrane was measured as described in Methods. MCF-7 cultured alone migrated through the transwell insert only after estradiol treatment, and such migration was blocked by tamoxifen or ICI 182,780 (Figure 1f, blue bars). In contrast, presence of conditioned KG-1 or THP-1 macrophages in the lower well resulted in migration of MCF-7 cells under all experimental conditions, including tamoxifen or ICI 182,780 treatment (Figure 1f, red bars).

**Figure 2:**
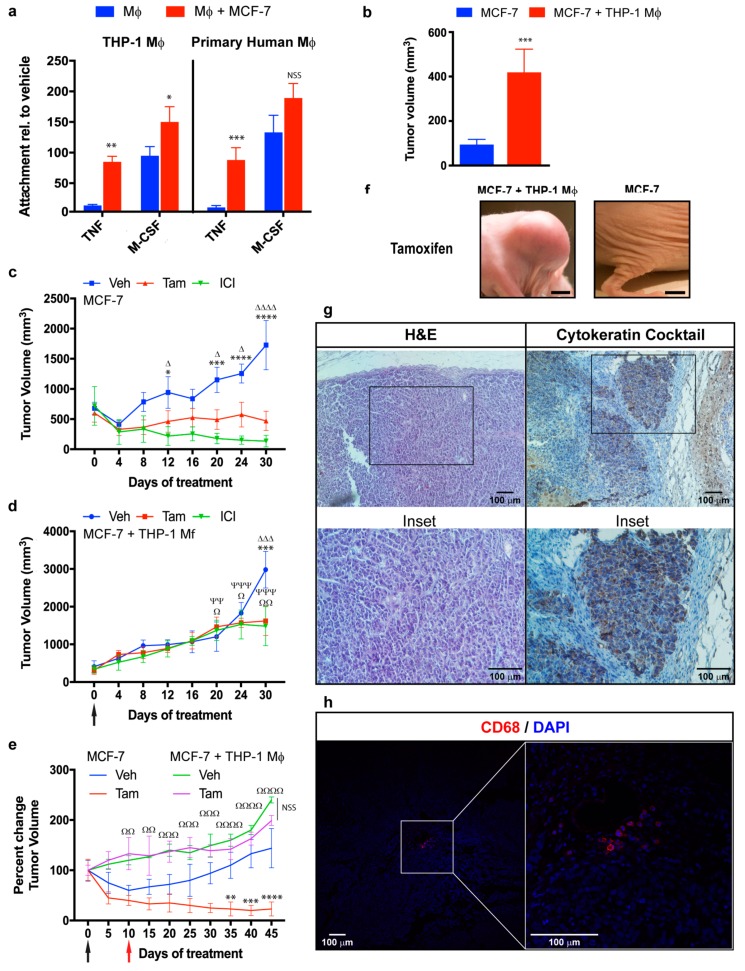
Macrophages induce MCF-7 xenograft tumor growth, which is not blocked by tamoxifen. (**a**) Differentiation-associated attachment of primary human or THP-1 monocytes (Mo) in the presence or absence of MCF-7. Mo were labeled with fluorophore, and fluorescence of attached cells was measured after 72 h M-CSF (10 ng/mL) or TNF-α (TNF) (1 ng/mL) treatment, relative to vehicle treatment. Data shown are mean fluorescence ± SEM from three independent experiments, *n* = 3. Analysis in comparison with absence of MCF-7. (**b**) Nude mice were implanted with 60-day slow release estradiol pellet, and injected in the right flank 24 h later with 1.2 × 10^6^ MCF-7, or 1.2 × 10^6^ MCF-7 plus 0.4 × 10^6^ THP-1. Data shown are mean ± SEM of tumor volumes 2 weeks after inoculation of MCF-7 (*n* = 37) or MCF-7 + THP-1 (*n* = 48). Analysis in comparison with absence of macrophages. (**c**,**d**) Tumor volumes of MCF-7 (**c**) and MCF-7/THP-1 xenografts (**d**). After tumor volume reached 500 mm^3^, animals were injected subcutaneously with vehicle (Veh) (peanut oil), tamoxifen (Tam) (10 mg/kg), or ICI 182,780 (ICI) (10 mg/kg) 4-day intervals. Black arrow: removal of estradiol pellet. Data shown are mean ± SEM (*n* = 8). * Veh vs. ICI; Δ Veh vs. Tam; Ω Tam (**d**) vs. Tam (**c**); Ψ ICI (**d**) vs. ICI (**c**). (**e**) Xenograft tumors generated from MCF-7 or MCF-7/THP-1 were treated with Veh (*n* = 8) or Tam (*n* = 9). Animals were injected subcutaneously at 5-day intervals. Black arrow: removal of estradiol pellet. Red arrow: re-implantation of estradiol pellet. * Veh vs. Tam (MCF-7); NSS Veh vs. Tam (MCF-7 + THP-1) or **Ω** Tam (MCF-7 + THP-1) vs. Tam (MCF-7). Values shown are mean change ± SEM, with initial tumor volume defined as 100%. (**f**) Representative photographs of MCF-7 and MCF-7/THP-1 xenograft tumors after 30 days tamoxifen treatment: Scale bars: 10 mm. (**g**) Left: Sections of xenograft tumors formed by MCF-7/THP-1 injection was stained with H&E and subjected to histological analysis. Right: CK7 expression was analyzed by IHC. Hematoxylin was used as nuclear counterstain. (**h**) CD68 expression was analyzed by IF in MCF-7/THP-1 xenograft tumor sections. Representative images are shown. Red: CD68 staining. Blue: DAPI staining of nuclei. * *p* < 0.05; ** *p* < 0.01; *** *p* < 0. 001; **** *p* < 0. 0001; Δ *p* < 0.05; ΔΔΔ *p* < 0.001; ΔΔΔΔ *p* < 0.0001; Ω *p* < 0.05; ΩΩ *p* < 0.01; ΩΩΩ *p* < 0.001; ΩΩΩΩ *p* < 0.0001; ΨΨ *p* < 0.01; ΨΨΨ *p* < 0.001; NSS: not statistically significant.

Breast cancer cells release various chemotactic factors (e.g., MCP-1) that attract monocytes from the bloodstream. Once at the tumor site, monocytes differentiate into macrophages under stimulation of factors such as M-CSF. We examined the possibility that monocyte differentiation is promoted by breast cancer cells when the two cell types are co-cultured. Differentiation of primary human or THP-1 monocytes, under TNF-α stimulation, was clearly enhanced by co-culture with MCF-7. Co-culture with MCF-7 also enhanced differentiation of THP-1 monocytes under M-CSF stimulation, whereas such effect was not significant in the case of primary human monocytes (Figure 2a).

To test the possibility that such promotion of monocyte differentiation also occurs in vivo, we placed a subcutaneous estradio pellet in immunocompromised mice and subsequently injected a suspension of MCF-7 cells and undifferentiated THP-1 monocytes, to mimic differentiation of circulating monocytes into macrophages by tumor cells. After two weeks, tumors derived from MCF-7/THP-1 co-injection were significantly (>3-fold) larger than those derived from injection of MCF-7 alone (Figure 2b). Injection of MCF-7 alone led to tumor formation in 35/50 mice (70%), whereas MCF-7/THP-1 co-injection led to tumor formation in 48/50 mice (96%). Progressive tumor growth in MCF-7-injected animals was inhibited by tamoxifen, and was reversed by ICI 182,780 (Figure 2c). In contrast, xenograft tumors generated by MCF-7/THP-1 co-injection continued growing regardless of tamoxifen or ICI 182,780 treatment, with doubling of volume by ~15 days (Figure 2d). In a standard MCF-7 xenograft model, tumor growth was blocked, removal of estradiol pellet caused tumor regression, and re-implantation of estradiol pellet on Day 10 caused resumption of tumor growth. Tamoxifen treatment in combination with estradiol pellet re-implantation inhibited tumor growth. In MCF-7/THP-1 co-injected mice, tumors continued growing after estradiol pellet removal, and there were no notable differences in groups treated with tamoxifen vs. vehicle (Figure 2e). Tumors from MCF-7/THP-1 co-injected mice treated with tamoxifen were CK7-positive, consistently with breast tumor lineage (Figure 2g, right panel). Hematoxylin/eosin staining revealed that the proportion of infiltrating leukocytes in these tumors was much lower than the initial ratio of MCF-7/THP-1 cells injected (4:1), indicating that the tumor mass consisted primarily of breast cancer cells (Figure 2g, left panel). Infiltrating leukocytes in tumors were associated mainly with human macrophages; IF revealed CD68-positivity, indicating that THP-1 monocytes differentiated in vivo (Figure 2h). These findings provide evidence for a role of macrophages in promoting endocrine resistance of breast cancer cells, including ER antagonist function and estrogen withdrawal, which mimics effects of aromatase inhibitors.

### 2.2. Modulation of Breast Cancer Proliferative Genes by Co-Culture with Macrophages

**Figure 3:**
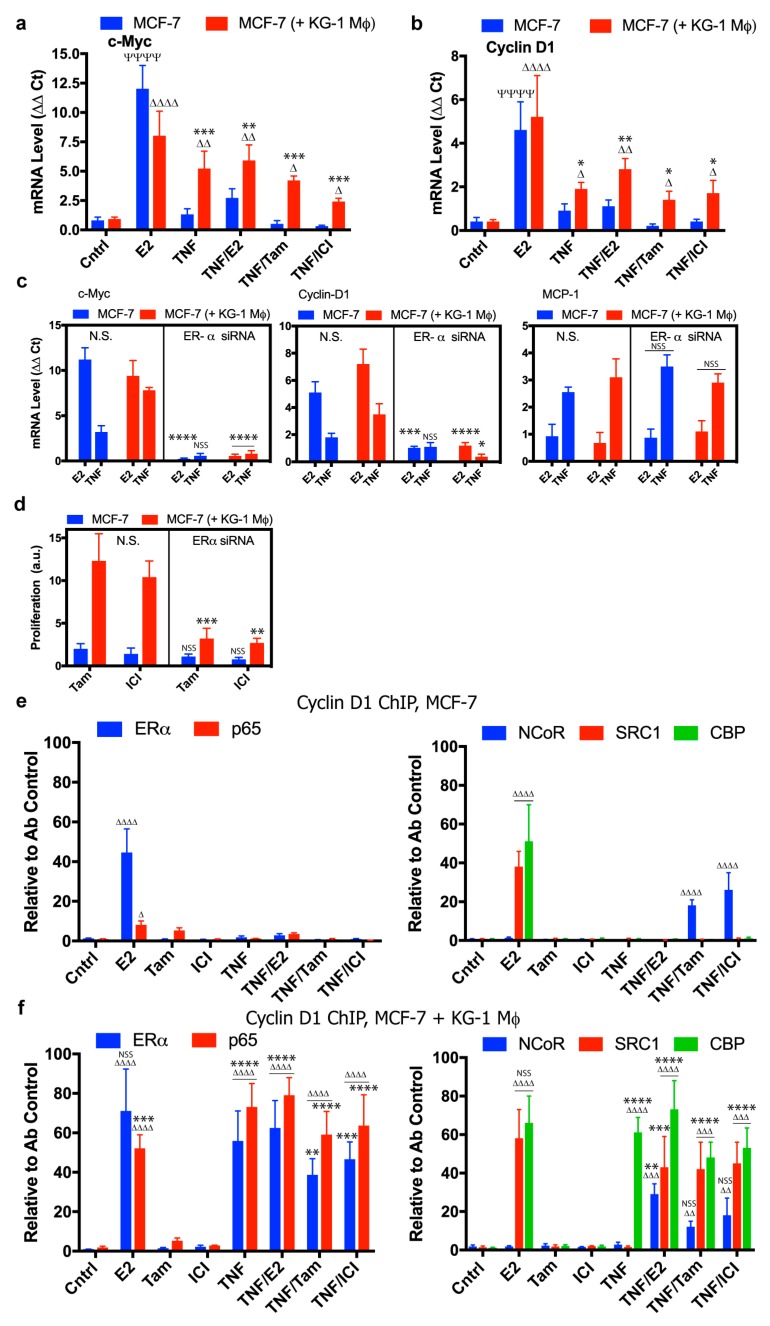
Altered expression of proliferative genes *c-Myc* and *cyclin D1*. (**a**,**b**) Expression levels of *c-Myc* (**a**) and *cyclin D1* (**b**) in MCF-7 cultured in presence or absence of conditioned KG-1 macrophages, following 2 h treatment with the indicated ligands. Relative mRNA level of each gene was quantified by qPCR with respect to control calculated by 2^−ΔΔCt^ method, *n* = 4. * in comparison with absence of macrophages in indicated treatment; Ψ or Δ in comparison with respective Cntrl of each group (blue: control MCF-7; red: control MCF-7/KG-1). (**c**) Expression levels of *c-Myc*, *cyclin D1*, and *MCP-1* in MCF-7 after 2 h treatment with indicated ligands. MCF-7 were previously transfected with ERα siRNA or non-silencing (N.S.) siRNA, and cultured for 2 days in the presence or absence of conditioned KG-1 macrophages. * in comparison with N.S. siRNA in indicated treatment of each group. (**d**) Proliferation of MCF-7 cultured in the presence or absence of KG-1 macrophages and stimulated with TNF-α (1 ng/mL). MCF-7 were transfected with ERα siRNA or N.S. siRNA and treated for two days with Tam or ICI and measured by CyQUANT, *n* = 3. (**e**,**f**) ChiP assay of ERα and p65 (left) or NCOR, SRC1, and CBP (right) followed by qPCR analysis of *cyclin D1* promoter in MCF-7 cultured alone (**e**) or with conditioned KG-1 macrophages (**f**) for 24 h, *n* = 4. The indicated treatments were applied 2 h prior to harvesting. Δ in comparison with Cntrl of each group; * in comparison with absence of macrophages in indicated treatment of each group; e.g., E2 (ERα MCF-7 + KG-1) vs. E2 (ERα MCF-7). Cntrl: Fresh DMEM, E2: Estradiol 1 nM, TNF: TNF-α 1 ng/mL, Tam: Tamoxifen 1 µM, ICI: ICI 182,780 1 µM. * *p* < 0.05; ** *p* < 0.01; *** *p* < 0. 001; **** *p* < 0. 0001; Δ *p* < 0.05; ΔΔ *p* < 0.01; ΔΔΔ *p* < 0.001; ΔΔΔΔ *p* < 0.0001; ΨΨΨΨ *p* < 0.0001; NSS: not statistically significant.

Expression of both cyclin D1 and c-Myc genes is required for estradiol-mediated proliferation in breast tumors (Figure S2a,b) [36]. These genes were both upregulated in E2-treated MCF-7 cells, but not in TNF-α-stimulated MCF-7 (Figure 3a,b, blue bars). In MCF-7 co-cultured with conditioned KG-1 macrophages, TNF-α stimulation was sufficient to induce c-Myc and cyclin D1 expression in the absence of estradiol (Figure 3a,b, red bars). Treatment with tamoxifen or ICI 182,780 only partially inhibited TNF-α-induced expression of these genes, suggesting that they are potential effectors of macrophage-mediated endocrine resistance. To test the idea that ERα is required for this response, we used siRNA to knock down ERα expression, thus blocking both estradiol- and macrophage-induced expression of c-Myc and cyclin D1, but not TNF-α-induced expression of MCP-1 (Figure 3c). ERα knockdown also blocked macrophage-mediated proliferation of TNF-α-treated MCF-7 exposed to tamoxifen or ICI 182,780 (Figure 3d). These findings indicate that macrophage-induced proliferation is ERα-dependent and associated with inappropriate activation of essential proliferative genes in breast cancer cells. Despite the ERα dependence, these effects were not completely blocked by tamoxifen or ICI 182,780, suggesting that ERα-mediated promotion of endocrine resistance by macrophages occurs in an estrogen-independent manner.

To elucidate the molecular mechanism whereby conditioned macrophages affect ERα+ breast cancer cells and induce estradiol-independent proliferation via TNF-α stimulation, we used chromatin cross-linking and ChIP assays to identify assembled protein complexes that operate on cyclin D1. In MCF-7 cultured alone, estradiol treatment promoted recruitment to the cyclin D1 promoter of transcription factor ERα and (to a lesser degree) NF-κB subunit p65 (Figure 3e, left panel). Coactivators CBP and SRC1 were also recruited under this condition. In contrast, TNF-α treatment did not induce recruitment of the above factors to the cyclin D1 promoter, but, in combination with tamoxifen or ICI 182,780 treatment, resulted in union with NCoR corepressor (Figure 3e, right panel). RNA polymerase (Pol) II and activated gene locus marker trimethylated Lys-4 on Histone-3 (Me3-H3K4) were also detected when cells were cultured with estradiol (Figure S2c).

Co-culture of MCF-7 with conditioned KG-1 macrophages greatly altered recruitment of protein complexes to cyclin D1 promoter. Estradiol stimulation notably increased level of p65 bound to cyclin D1 promoter, while recruitment of ERα, CBP, and SRC1 was maintained (Figure 3f, left and right panels). TNF-α stimulation of co-cultured MCF-7 induced recruitment of ERα and p65 at high levels, together with cofactor CBP, at the cyclin D1 promoter, even in the absence of estradiol (Figure 3f, left and right panels). Pol II binding and Me3-H3K4 marker level increased, indicating that the gene is transcriptionally active under this condition (Figure S2c). Tamoxifen or ICI 182,780 treatment did not prevent TNF-α-induced recruitment of ERα or p65 at cyclin D1 promoter of co-cultured MCF-7 (Figure 3f, left panel). Treatment with ERα antagonists primarily affected cofactors that constitute the transcriptional complex; i.e., union of NCoR corepressor was induced, and SRC1 and CBP coactivators were recruited concurrently (Figure 3f, right panel). NCoR recruitment at cyclin D1 promoter resulting from tamoxifen or ICI 182,780 treatment did not prevent binding of Pol II or Me3-H3K4 marker (Figure S2c). Findings for c-Myc promoter were nearly identical (Figure S3a–f), indicating that the processes of macrophage-mediated proliferation and endocrine resistance in breast cancer cells are associated with differential recruitment of transcription factors ERα and NF-κB, and of transcriptional coactivators, to promoters of genes that play key roles in cell cycle progression.

### 2.3. Role of NF-κB in Macrophage-Mediated MCF-7 Proliferation

**Figure 4:**
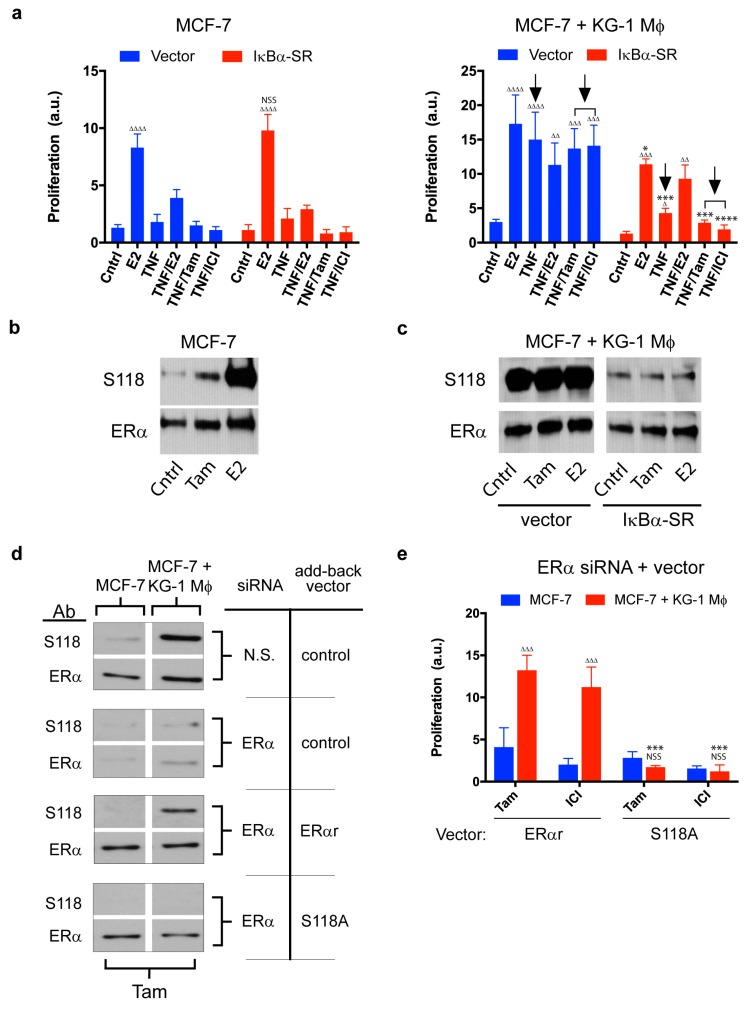
Role of NF-κB in macrophage-mediated breast cancer cell proliferation and ERα phosphorylation. (**a**) Proliferation of MCF-7 transfected with IκBα super-repressor (IκBα-SR), or with empty vector as control, and incubated in presence of conditioned KG-1 macrophages or in their absence for 48 h with the indicated treatments. Data shown are expressed in arbitrary units (a.u.), *n* = 3. Arrows indicate how the treatment is affected with IκBα-SR. Δ in comparison with the respective Cntrl of each group; * in comparison with the same treatment in cells expressing or not the IκBα-SR within each group (MCF-7 or MCF-7 + KG-1 Mφ). (**b**) Western blotting (WB) of total ERα or phospho-S118 ERα (S118) from MCF-7 treated with the indicated ligands for 2 h prior to cell harvesting. (**c**) WB of total ERα or phospho-S118 ERα (S118) from MCF-7 co-cultured with conditioned KG-1. MCF-7 were transfected with empty vector or with the IκBα-SR and cultured with the indicated ligands for 2 h prior to harvesting. (**d**) WB of total ERα or phospho-S118 ERα (S118) from MCF-7 or MCF-7 co-cultured with conditioned KG-1. MCF-7 were transfected with a vector expressing a refractory ERα protein identical to the endogenous (ERαr), a mutated variant of the ERαr (S118A) or with the empty vector. Then, cells were treated with an siRNA targeting the endogenous ERα or N.S. control. Cells were treated with Tam for 2 h prior to cell harvesting. (**e**) Proliferation of MCF-7 in the presence or the absence of conditioned KG-1 separated by a semipermeable membrane was measured after two days of culture, *n* = 3. MCF-7 expressing ERαr or ERαr S118A were treated with siRNA targeting endogenous ERα. MCF-7 cultured alone were stimulated with E2 and those co-cultured with conditioned macrophages with TNF. Δ in comparison with absence of macrophages in indicated treatment of each group; * in comparison with Vector ERαr in indicated treatment of each group. Cntrl: Fresh DMEM, E2: Estradiol 1 nM, TNF: TNF-α 1 ng/mL, Tam: Tamoxifen 1 µM, ICI: ICI 182,780 1 µM. * *p* < 0.05; *** *p* < 0. 001; **** *p* < 0. 0001; Δ *p* < 0.05; ΔΔ *p* < 0.01; ΔΔΔ *p* < 0.001; ΔΔΔΔ *p* < 0.0001; NSS: not statistically significant.

To test the hypothesis that NF-κB signaling is required for macrophage-mediated endocrine resistance, we transfected MCF-7 cells with control vector or a vector expressing dominant negative IκBα super-repressor, which prevents NF-κB relocalization into the nucleus. IκBα super-repressor expression affected proliferation rate of MCF-7 cocultured with conditioned KG-1 macrophages (separated by semipermeable membrane), but not of MCF-7 cultured alone (Figure 4a). This finding suggests that NF-κB plays a key role in signaling pathways involved in macrophage-induced proliferation, but not in estradiol-mediated canonical proliferation. It was surprising that behavior of co-cultured MCF-7 expressing IκBα super-repressor was similar to that of MCF-7 cultured alone. IκBα overexpression affected all tested variables under the cocultured condition. In the presence of E2, the proliferation rate of co-cultured MCF-7 was similar to that of MCF-7 alone. IκBα strongly inhibited proliferation of co-cultured MCF-7 treated with TNF-α, tamoxifen, or ICI 182,780 (Figure 4a, right panel, arrows). These findings, taken together, indicate that macrophage-mediated endocrine resistance of breast cancer cells requires an intact NF-κB pathway.

We have shown that transcription factors NF-κB and ERα are both essential for estradiol-independent MCF-7 proliferation. To elucidate the mechanism whereby NF-κB affects ligand-independent activation of ERα by macrophages, we examined Ser-118 phosphorylation status in ERα. This modification is associated with clinical tamoxifen resistance, ligand-independent ERα activation, and enhanced coactivator recruitment to ERα response elements of target gene promoters [25]. In MCF-7 cultured alone, ERα Ser-118 phosphorylation was observed following estradiol treatment, and this effect was significantly reduced by tamoxifen (Figure 4b). In contrast, in co-cultured MCF-7, ERα Ser-118 phosphorylation was observed in the absence of E2 and even in the presence of tamoxifen. Such phosphorylation was significantly reduced by IκBα super-repressor, indicating that it is dependent on the NF-κB pathway (Figure 4c).

The role of ERα Ser-118 in macrophage-mediated proliferation was evaluated by experiments combining siRNA for endogenous ERα and a mutant refractory to ERα siRNA knockdown (ERαr). ERα siRNA effectively blocked expression of endogenous ERα protein in MCF-7 (Figure 4d), and transfection of the ERαr vector restored macrophage-mediated ERα S118 phosphorylation and proliferation in the presence of tamoxifen (Figure 4d,e). Expression of ERαr S118A with a disrupted phosphorylation site (to a degree similar to that of endogenous ERα protein) failed to restore SERM-resistant proliferation of co-cultured MCF-7 (Figure 4e). These findings indicate that NF-κB-mediated signaling pathway induces ERα phosphorylation, facilitating ligand-independent proliferation as observed in co-cultured MCF-7. Also, we know that more study is necessary to know the precise mechanism by which NF-κB-mediated signaling induces ERα phosphorylation.

### 2.4. Role of the IL-6/STAT3 Pathway in Macrophage-Mediated MCF-7 Endocrine Resistance

Macrophage-mediated endocrine resistance occurs without direct contact between the two cell types. To evaluate possible involvement of cytokines in effects of macrophages on ER+ breast cancer cells, we incubated a cytokine antibody array with CM collected from supernatants of: (i) MCF-7 cultured alone; (ii) KG-1 macrophages cultured alone; (iii) MCF-7 co-cultured with KG-1; (iv) MCF-7 co-cultured with KG-1 in the presence of tamoxifen. In each case, cell culture was initially stimulated for 6 h with 1 ng/mL TNF-α, and cells were then washed and cultured in fresh medium for 24 h without addition of factors. To facilitate interpretation of data, we plotted the ratio of cytokine level of CM from co-cultured cells relative to the sum of cytokine levels of CM from individual cultures. In co-culture experiments with 79 cytokines, the ratio increased (value >1) in 59% of cases, decreased (value <1) in 32% of cases, and remained unchanged (value ~1) in 9% of cases, reflecting the complexity of these interactions (Figure S4). The changes in expression of these cytokines were due to interactions between macrophages and ER+ breast cancer cells. Upregulation of several of the cytokines (including IL-6, MCP-1, CCL5, IL-8, and IL-1α) was not inhibited by tamoxifen.

**Figure 5:**
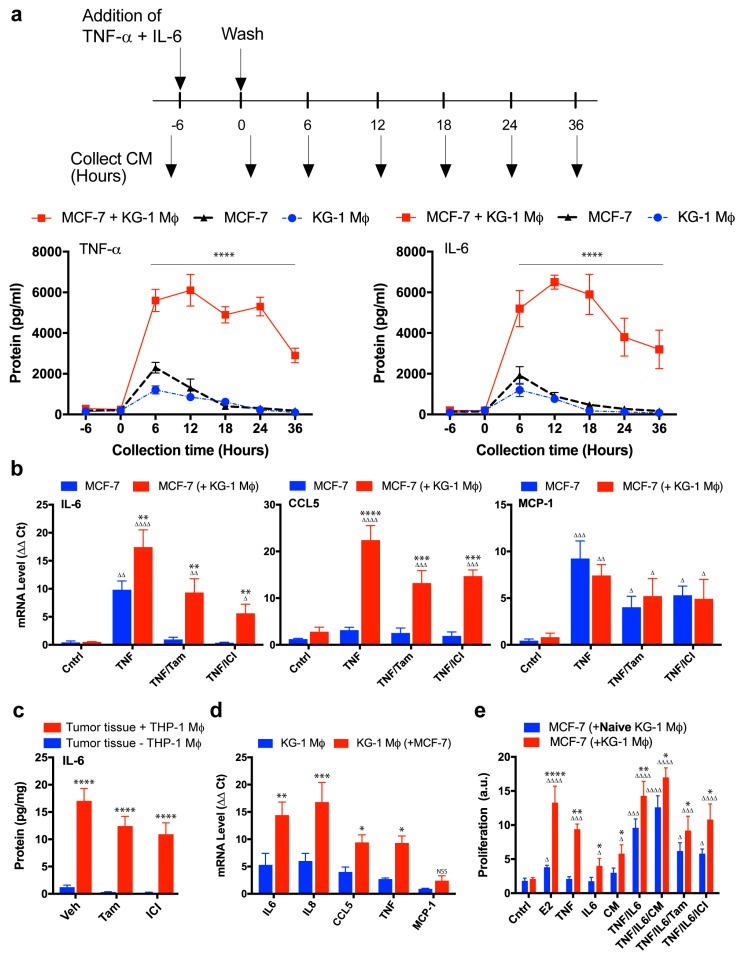
Role of IL-6 in macrophage mediated endocrine resistance. (**a**) Timeline of cell treatment: MCF-7, KG-1 macrophages or MCF-7 co-cultured with macrophages were treated with TNF and IL-6 for 6 h and then washed. CM was collected as indicated with the lower arrows every 6 h during 36 h and then assayed by ELISA. Graphics show the level of TNF-α and IL-6 in CM from the different cell cultures, *n* = 5. (**b**) *IL-6*, *CCL5*, and *MCP-1* mRNA expression from MCF-7 cells cultured in the presence or the absence of conditioned KG-1 macrophages separated by a semipermeable membrane and treated with the indicated ligands for 2 h prior to harvesting. Mean gene expression was calculated by the 2^−ΔΔCt^ method, *n* = 4. Δ in comparison with the Cntrl of each group; * in comparison with absence of macrophages in indicated treatment. (**c**) IL-6 protein expression from xenograft tumors of MCF-7 cells grown, with or without THP-1 cells, for 30 days was measured by quantitative ELISA. Protein expression is expressed as pg of IL-6 protein per mg of tumor tissue, *n* = 6. Analysis in comparison with the absence of macrophages in tumor. (**d**) Conditioned KG-1 macrophages were cultured in transwell with or without MCF-7 cells separated by a semipermeable membrane, following 2 h treatment with TNF the expression of the indicated genes was measured in the macrophages with respect to its control (unstimulated cells) by the 2^−ΔΔCt^ method, *n* = 4. Analysis in comparison with the absence of MCF-7 in indicated treatment. (**e**) Proliferation of MCF-7 plus naïve KG-1 macrophages or conditioned KG-1 macrophages, separated by a semipermeable membrane after 48 h of the indicated treatments. Proliferation was measured by CyQUANT, *n* = 5, and expressed in arbitrary units (a.u.). Δ in comparison with Cntrl of each group; * in comparison with the presence of naïve macrophages in indicated treatment. Cntrl: Fresh DMEM, E2: Estradiol 1 nM, TNF: TNF-α 1 ng/mL, CM: Conditioned media 10%, IL-6: Interleukin 6 1 ng/mL, Tam: Tamoxifen 1 µM, ICI: ICI 182,780 1 µM. * *p* < 0.05; ** *p* < 0.01; *** *p* < 0. 001; **** *p* < 0. 0001; Δ *p* < 0.05; ΔΔ *p* < 0.01; ΔΔΔ *p* < 0.001; ΔΔΔΔ *p* < 0.0001; NSS: not statistically significant.

We decided to validate these findings for IL-6 and TNF-α at various times, since IL-6 was the proinflammatory cytokine displaying the greatest increase. TNF-α is able to induce IL-6 expression through NF-κB, and we have demonstrated the crucial role of TNF-α in induction of proliferation, migration, and endocrine resistance in co-cultured MCF-7. MCF-7 and KG-1 were cultured alone, or co-cultured with separation by a semipermeable membrane as described in Methods. Cells were stimulated for 6 h with TNF-α and IL-6, washed, and medium was replaced with fresh medium. Samples of culture medium were taken every 6 h, and TNF-α or IL-6 protein level was analyzed by ELISA. TNF-α and IL-6 levels were higher in co-cultured cells than in either MCF-7 or KG-1 cultured alone (Figure 5a). TNF-α and IL-6 levels were maintained for 24 h in co-culture because of interaction between the two cell types, whereas levels in the single cultures declined over time, reaching basal values by 24 h.

Expression levels of various genes that normally respond to NF-κB were analyzed in MCF-7. TNF-α stimulation induced expression of IL-6 and MCP-1 but not of CCL5 (Figure 5b). In co-cultured MCF-7, TNF-α stimulation greatly increased expression levels of CCL5 and IL-6, but not of MCP-1. Co-culture of MCF-7 also inhibited modulation of CCL5 and IL-6 expression by tamoxifen or ICI 182,780 treatment. Consistently with these findings, intratumoral human IL-6 protein levels in tumors of mice co-injected with MCF-7 cells and THP-1 monocytes were significantly higher than in tumors formed from MCF-7 alone. Tamoxifen or ICI 182,780 treatment had no effect on intratumoral IL-6 protein level (Figure 5c).

To evaluate the effects of breast cancer cells on cytokine production in macrophages, we cultured conditioned KG-1 alone or with MCF-7 (separated by semipermeable membrane) for 24 h. Following TNF-α stimulation, transcription levels of genes encoding IL-6, IL-8, CCL5, and TNF-α (but not MCP-1) were much higher in co-cultured KG-1 than in KG-1 alone (Figure 5d).

To evaluate the possibility (suggested by the above findings) that IL-6 and TNF-α in combination are required for promotion of macrophage-mediated endocrine resistance, we measured proliferation of MCF-7 co-cultured with naïve or conditioned KG-1 and treated with TNF-α and/or IL-6. Treatment with TNF-α or IL-6 alone did not induce proliferation of co-cultured MCF-7/naïve KG-1 (Figure 5e), but did induce proliferation of co-cultured MCF-7/conditioned KG-1. The effect of TNF-α was significantly greater than that of IL-6. Treatment with TNF-α and IL-6 in combination (TNF-α/IL-6) induced proliferation of MCF-7 co-cultured with either naïve or conditioned KG-1. Proliferation rate was always greater for MCF-7 co-cultured with conditioned than with naïve KG-1. Tamoxifen or ICI 182,780 treatment had no effect on TNF-α/IL-6-induced proliferation of MCF-7 even when co-cultured with naïve KG-1. These findings indicate that TNF-α/IL-6 are necessary and sufficient for induction of macrophage-mediated MCF-7 proliferation and endocrine resistance, although other cytokines may conceivably be involved in the complex interaction network between breast cancer cells and macrophages. Proliferation rate of co-cultured MCF-7/naïve KG-1 treated with TNF-α/IL-6 was slightly greater in the presence vs. the absence of CM (Figure 5e). Such CM, obtained from co-cultured MCF-7/conditioned KG-1 (see Methods), contained the complete, complex mixture of factors released into culture medium as a result of interaction between the two cell types (Figure S4).

The above findings indicate that co-culture of MCF-7 with macrophages generates an inflammatory-prone gene expression profile that promotes resistance to the suppressive effects of ER antagonists. In view of the well-documented functions of the IL-6/STAT3 signaling pathway in tumor growth promotion [60,68,69], we examined its role in macrophage-mediated breast cancer cell proliferation.

**Figure 6:**
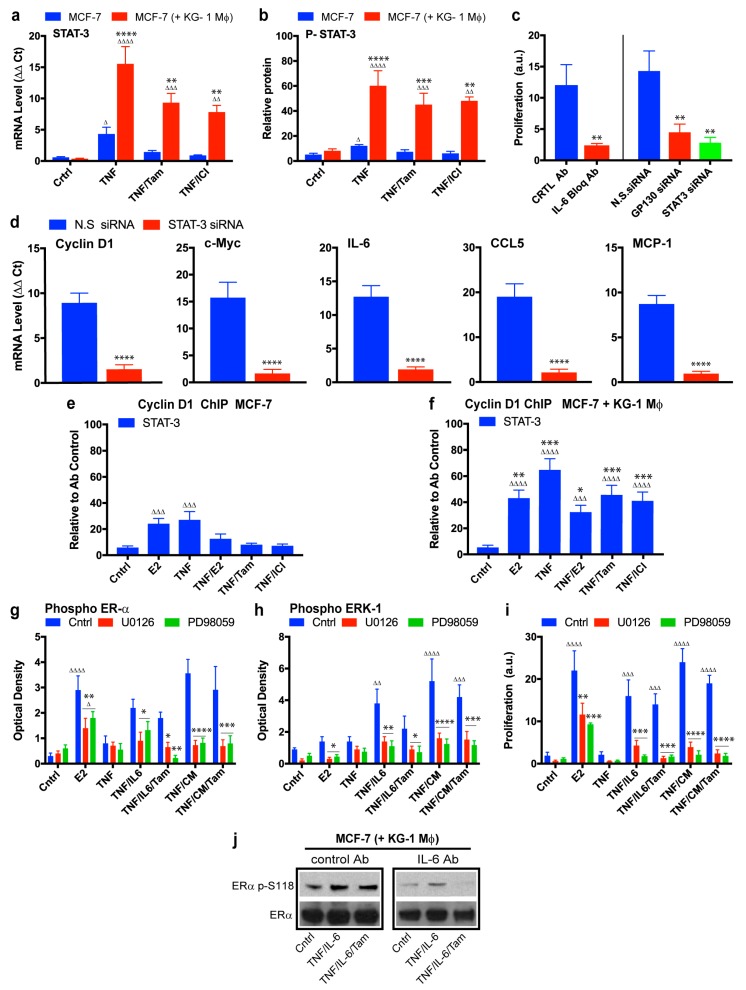
Role of IL-6/STAT3 in macrophage-mediated endocrine resistance. (**a**) STAT3 mRNA expression from MCF-7 cells cultured in the presence or the absence of conditioned KG-1 macrophages and treated with the indicate ligands for 2 h prior to cell harvesting. Relative mRNA expression with respect to control was calculated by the 2^−ΔΔCt^ method, *n* = 4. Δ in comparison with Cntrl of each group; * in comparison with absence of macrophages in indicated treatment; e.g., TNF (MCF-7+ Macrophages) vs. TNF (MCF-7). (**b**) Content of p-STAT3 protein in CM from the culture of MCF-7 or the co-culture of MCF-7 with conditioned KG-1 macrophages, treated with the indicated ligands for 2 h prior to harvesting and assayed with quantitative ELISA. Results are the mean relative content with respect to Cntrl. Δ in comparison with Cntrl of each group; * in comparison with absence of macrophages in indicated treatment; e.g., TNF (MCF-7+ Macrophages) vs. TNF (MCF-7). (**c**) Proliferation of MCF-7 co-cultured with conditioned KG-1 macrophages and treated with TNF plus Tam. Four h prior to the TNF/Tam treatment, MCF-7 were incubated as indicated in each case. Non-specific IgG antibody (Cntrl Ab) and N.S. siRNA were used as respective controls. Analysis in comparison with the respective control: Cntrl Ab or N.S. siRNA. (**d**) MCF-7 were transfected with either N.S. (blue bar) or STAT3 targeted siRNA (red bar). Following 48 h MCF-7 were co-cultured with conditioned KG-1 macrophages overnight and treated with TNF plus Tam for 2 h before processing for qPCR. Relative mRNA expression of (left to right) cyclin D1, c-Myc, IL-6, CCL-5 or MCP-1 was calculated by the 2^−ΔΔCt^ method, *n* = 4. Unstimulated MCF-7 treated with N.S. siRNA was used as control. Analysis in comparison with N.S. siRNA of each group (blue bar). (**e**,**f**) ChiP assay of STAT3 followed by qPCR analysis of the *cyclin D1* promoter in MCF-7 cells cultured alone (e) or in the presence of conditioned KG-1 macrophages (f) for 24 h. The cultures received the indicated treatments 2 h prior to harvesting. Results are normalized to non-specific IgG Ab, *n* = 4. Δ in comparison with Cntrl of each group; * in comparison with absence of macrophages in indicated treatment of each group; e.g., E2 (ERα MCF-7+ Macrophages) vs. E2 (ERα MCF-7). (**g**,**h**) phospho-ERα (**g**) and Phospho-ERK-1 (**h**) protein expression from MCF-7 cultured in the absence of macrophages. MCF-7 were treated with specific ERK pathway inhibitors (U0126 and PD98059) 2 h before being treated as indicated by another 2 h. Determination of protein expression was performed by quantitative ELISA. *n* = 4. Δ in comparison with Cntrl of each group. * in comparison with the presence of inhibitors (U0126 and PD98059) versus Cntrl in indicated treatment; e.g., E2 (UO126, red bar) vs. E2 (cntrl, blue bar). (**i**) Proliferation of MCF-7 cultured in the absence of macrophages for 2 days with or without ERK pathway inhibitors and the indicated ligands. Proliferation was measured by CyQUANT, *n* = 3, and expressed in arbitrary units (a.u.). Δ in comparison with Cntrl of each group. * in comparison with presence of inhibitors (U0126 and PD98059) versus Cntrl in indicated treatment; e.g., E2 (UO126, red bar) vs. E2 (cntrl, blue bar). (**j**) Western blotting of phospho-S118 ERα (S118) or total ERα from MCF-7 which were co-cultured with conditioned KG-1 macrophages and treated with the indicated ligands for 2 h prior to cell harvesting. IL-6 blocking Ab or control Ab were added to the culture media 4 h prior to harvesting. Cntrl: Fresh DMEM, E2: Estradiol 1 nM, TNF: TNF-α 1 ng/mL, CM: Conditioned media 10%, IL-6: Interleukin 6 1 ng/mL, Tam: Tamoxifen 1 µM, ICI: ICI 182,780 1 µM. * *p* < 0.05; ** *p* < 0.01; *** *p* < 0. 001; **** *p* < 0. 0001; Δ *p* < 0.05; ΔΔ *p* < 0.01; ΔΔΔ *p* < 0.001; ΔΔΔΔ *p* < 0.0001.

In MCF-7 cultured alone, TNF-α induced STAT3 expression, and such induction was blocked by either tamoxifen or ICI 182,780 (Figure 6a, blue bars). In contrast, TNF-α induced STAT3 expression was much higher in co-cultured MCF-7, and such increase was not downregulated by tamoxifen or ICI 182,780 (Figure 6a, red bars). To confirm this finding at the protein level, we performed ELISA analysis of phospho-STAT3 induction in MCF-7 under the same conditions. Variations in phospho-STAT3 levels were correlated with those of STAT3 mRNA. Macrophages induced TNF-α-dependent increase of phospho-STAT3 in MCF-7, and such upregulation was not blocked by tamoxifen or ICI 182,780 (Figure 6b). Thus, NF-κB-dependent gene expression, including IL-6/STAT3 signaling pathway, was significantly enhanced by conditioned macrophages, and such enhancement was not blocked by tamoxifen. To examine possible interactions between ERα and STAT3 pathways, we transfected MCF-7 with ERα siRNA and evaluated STAT3 mRNA expression levels under various conditions. ERα downregulation had no effect on STAT3 mRNA expression level under any condition (Figure S5a). We demonstrated similarly that STAT3 mRNA downregulation had no effect on ERα mRNA expression level (Figure S5b).

We evaluated the role of IL-6/STAT3 signaling pathway in conditioned macrophage-induced MCF-7 proliferation by inhibiting IL-6 receptor and by blocking STAT3 expression. MCF-7 were treated with IL-6 receptor-blocking antibody or with siRNA targeting gp130 (a subunit of IL-6 receptor) or STAT3. MCF-7 proliferation was significantly reduced by each of these treatments (Figure 6c), strongly suggesting involvement of the IL-6/STAT3 pathway in non-canonical, estradiol-independent, conditioned macrophage-induced MCF-7 proliferation. STAT3 directly stimulates cyclin D1 and c-Myc expression [35,70,71], and indirectly stimulates a subset of NF-κB-dependent genes (including IL-6, CCL5, and IL-8) by tethering to NF-κB protein [72]. siRNA knockdown of STAT3 blocked macrophage-induced expression of breast cancer proliferative genes cyclin D1 and c-Myc, and of cytokines IL-6, CCL5, and MCP-1 (Figure 6d), demonstrating the essential role of STAT3 expression in conditioned macrophage-induced alteration of gene expression.

We next examined the association status of STAT3 with cyclin D1 promoter in MCF-7 cultured in the presence or absence of conditioned KG-1. ChIP assay revealed that in MCF-7 cultured alone STAT3 was associated with cyclin D1 promoter through estradiol or TNF-α stimulation, and that tamoxifen or ICI 182,780 treatment blocked such TNF-α-induced association (Figure 6e). Estradiol- or TNF-α-induced STAT3 recruitment to cyclin D1 promoter was higher for co-cultured MCF-7 than for MCF-7 alone, and tamoxifen or ICI 182,780 treatment did not block TNF-α-induced association of STAT3 with cyclin D1 promoter (Figure 6f).

We examined the relationship between IL-6/STAT3 pathway and ERα phosphorylation status at Ser-118. TNF-α/IL-6 stimulation of MCF-7 cultured alone resulted in increased phospho-ERα protein level, even in the presence of tamoxifen. Phospho-ERα levels under CM stimulation were slightly higher than those under TNF-α/IL-6 stimulation, demonstrating the complexity of the phenomenon (Figure 6g, blue bars). ERK-1 was evaluated as a kinase potentially involved in TNF-α/IL-6-induced ERα phosphorylation, since such stimulation induced ERK-1 activation (phospho-ERK-1) even in the presence of tamoxifen (Figure 6h, blue bars). MCF-7 were treated with two ERK-1 kinase inhibitors (U0126 and PD98059) to prevent ERK-1 phosphorylation (Figure 6h, red and green bars). Such inhibition of ERK-1 kinase strongly inhibited TNF-α/IL-6-induced phospho-ERα level increase, indicating that this pathway controls ER activation in estradiol-independent manner (Figure 6g, red and green bars).

MCF-7 proliferation under treatment with ERK-1 inhibitors was examined to evaluate the biological relevance of ERK-1. Treatment with ERK-1 inhibitors blocked TNF-α/IL-6-induced or CM-induced MCF-7 proliferation, but only partially reduced estrogen-induced proliferation (Figure 6i). Conditioned macrophage-induced S118 ERα phosphorylation in the presence of tamoxifen was inhibited by IL-6-blocking antibody treatment (Figure 6j), suggesting that NF-κB-mediated IL-6 induction could be the responsible for S118 ERα phosphorylation and activation. These findings, taken together, indicate that macrophage-mediated MCF-7 endocrine resistance depends on NF-κB and its induction of the IL-6 pathway.

## 3. Discussion

A variety of mechanisms whereby breast tumors acquire resistance to tamoxifen treatment have been studied. These mechanisms include changes in expression and/or post-translational modifications of ER, alterations in coregulatory proteins, increased AP-1 activity, and cell cycle deregulation [73,74,75,76,77]. Evidence to date suggests that many of the mechanisms of tamoxifen resistance involve increased signaling of receptor tyrosine kinases (e.g., EGFR, HER-2, and IGF-1R kinases), leading to activation of ERK and PI3K pathways [78]. Breast cancer cell line MCF-7 is ERα+ and responsive to treatment with SERMs and pure ER antagonists. Results of the present study indicate a mechanism whereby MCF-7 cells that interact paracrinically with conditioned macrophages proliferate following TNF-α stimulation, and display resistance to tamoxifen or ICI 182,780 treatment. Similar effects are observed in other ER+ breast cancer cell lines, but not in ER- cell lines (Figure 1).

Macrophages are a type of cells with high plasticity and the ability to activate a variety of functional programs depending on signals they receive from their environment. Multiple different populations of tumor-associated macrophages (TAMs) may coexist in a tumor, depending on the microenvironment [79]. TAMs typically have profiles similar to those of M2 (a.k.a. alternative) macrophages, which are involved in wound healing and tissue growth. TAMs often display high constitutive expression of IL-1β, IL-6, IL-8, and TNF-α [80]. High levels of TAMs infiltrated in breast tumors are correlated with worse clinical prognosis [81]. TAMs play various roles in cancer development and progression; e.g., they may promote tumor cell growth, remodeling of extracellular matrix, invasion of surrounding tissue, formation of metastatic deposits, and local immunosuppression [51]. Along this line, conditioned macrophages in the present study were found to promote in vitro proliferation, migration, and colony formation of breast cancer cells, and in vivo tumor development (Figure 1 and Figure 2). Few studies to date have addressed the role of TAMs in development of endocrine resistance [57,82]. We found that xenograft tumors from animals injected with MCF-7 cells did not grow following removal of estradiol pellet, but did grow when macrophages were co-injected, demonstrating the contribution of macrophages to development of endocrine resistance in vivo (Figure 2). Both endocrine and inflammatory signaling pathways in breast cancer cells were strongly affected by macrophages. Co-culture of MCF-7 with conditioned KG-1 resulted in sustained release of TNF-α and IL-6 from both cell types (Figure 5a), and consequent activation of NF-κB, STAT3, and ERα in MCF-7. NF-κB, STAT3, and c-Myc are involved in polarization of macrophages toward M2 profile, which is associated with pro-tumoral activity [83,84,85]. We observed that the co-culture of conditioned THP-1 macrophages with MCF-7 increased the expression of the M2 marker CD206 and did not produce significative changes in the expression of the M1 marker CD86, but conditioned macrophages showed greater expression level of CD86 than the control. These results suggest that co-culture with MCF-7 induce subpopulation of macrophages (Figure S6). By convention macrophage subpopulations have been described as having proinflammatory and tumoricidal capacities, classified as “M1” (classically activated), or those classified as "M2" (alternatively activated) specialized in suppressing inflammation, pro-tumor activities and repairing damaged tissues. Although the M1/M2 dichotomy provides convenience, this system does not represent the complex functional spectrum acquired in response to complex and changing stimuli. In addition, macrophages activated in classical and alternatively form represent states in a continuum, where genetic and molecular characteristics are not mutually exclusive. Therefore, the classification of subpopulations of macrophages in mammary tumors by source/anatomical location, the stimulating agent and the specific phenotype by the defined transcription factor and/or combinations of cell surface markers should be carefully considered. Rather, we should classify them as a whole by their function (pro-tumor or antitumor) since it is a very complex process [52]. Our findings indicate that both TNF-α and IL-6 are essential for macrophage-mediated induction of breast cell proliferation and endocrine resistance. TNF-α/IL-6 treatment was sufficient to induce proliferation of MCF-7 co-cultured with naïve macrophages, and proliferation rate was further increased by presence of macrophage CM (Figure 5d), suggesting that other factors are also involved in this complex interaction. E2-independent proliferation of MCF-7 was dependent on transcription factors ERα, NF-κB, and STAT3; absence of these factors significantly reduced TNF-α-induced proliferation in the presence of conditioned macrophages (Figure 3a, Figure 4a and Figure 6c).

Ligand-independent phosphorylation of ERα, which renders it constitutively active, is an important factor in conditioned macrophage-mediated development of MCF-7 endocrine resistance. Cascades of various types of kinase, including PKA (protein kinase A), ERK-1, and PAK (P21-activated kinase), are associated with tamoxifen resistance. These kinases induce phosphorylation of ERα or its cofactors [78,86,87]. We found that MCF-7 having the ERα S118A point mutation did not proliferate upon treatment with TNF-α and tamoxifen in the presence of conditioned macrophages (Figure 4d,e). This finding suggests that ERα phosphorylation at Ser-118 is essential for E2-independent MCF-7 proliferation, although we cannot rule out the possibility that other ERα residues are phosphorylated during this process. ERK-1 is evidently involved in ERα phosphorylation at Ser-118, since two specific ERK-1 inhibitors (U0126, PD98059) blocked ERα phosphorylation and consequent conditioned macrophage-mediated endocrine resistance (Figure 6g–i). ERK-1 is activated by both TNF-α and IL-6 pathways, but can also be activated by IL-8, another of the cytokines found to be increased during MCF-7/KG-1 co-culture (Figure S4) [88]. Along this line, tamoxifen-resistant MCF-7 were recently shown to express high levels of IL-8 [89]. In ovarian cancer cells, IL-6 and IL-8 activated estradiol-responsive genes, and reciprocal regulation between these signaling pathways was observed [90], similarly to our findings in breast cancer cells.

ChIP assay showed that TNF-α stimulation of co-cultured MCF-7/conditioned macrophages induced binding of p65, ERα, and STAT3 to cyclin D1 promoter (Figure 3e,f and Figure 6e,f). This finding suggests that these proteins may be components of a common transcriptional complex (of course, other proteins could be components of such complex as well). IP followed by Western blotting showed that interaction of these three proteins was strongly enhanced in co-cultured MCF-7/conditioned KG-1, even under tamoxifen treatment (Figure S7). The disposition of p65, ERα, and STAT3 in such transcriptional complex cannot be clearly defined at this point. However, in view of observations that part of the amplified cyclin D1 promoter has an NF-κB binding site [91,92], and that the ERα phosphorylated at Ser-118 can bind indirectly to DNA via transcription factors AP-1, SP-1, or NF-κB in a ligand-independent manner [27,93], it is conceivable that p65 can bind directly to DNA via NF-κB, with ERα acting as cofactor. Clear variation in recruitment of CBP and SRC1 coactivators and NCoR corepressor was observed, along with the transcriptional complex described in cyclin D1 promoter. Tamoxifen-ERα complex has been reported to exert antitumor activity through binding of NCoR to promoters of ERα target genes [94]. Although tamoxifen treatment induced recruitment of NCoR to cyclin D1 promoter in the present study, it did not block cyclin D1 expression (Figure 3b), and MCF-7 proliferated under these conditions (Figure 1a,b). Increased levels of SRC1 and CBP coactivators, both of which have histone acetyltransferase capacity, may account for observation of a transcriptionally active gene at the same time that NCoR corepressor is bound. NCoR has no intrinsic repressive capacity; rather, its function depends on recruitment of complexes that contain histone deacetylases. SRC-1 has been shown to induce endocrine resistance in breast cancer [95], and is considered an independent clinical predictor of worse prognosis [96].

**Figure 7:**
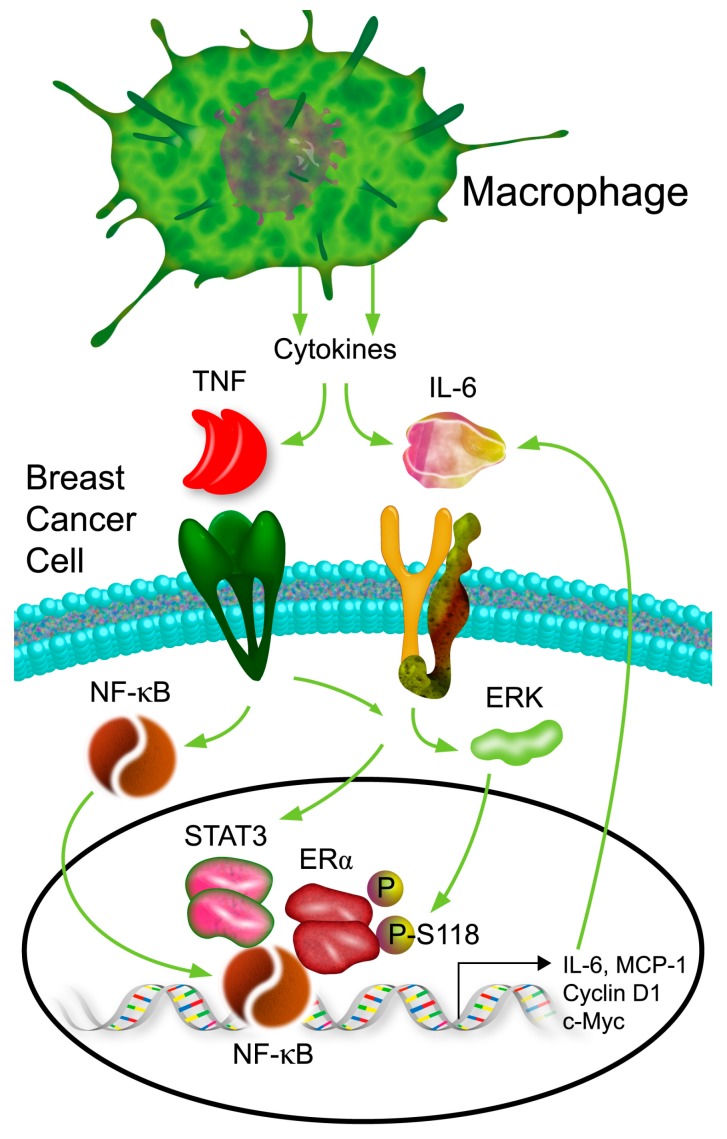
Schematic model of macrophage effects on ERα+ breast cancer cells. Endocrine resistance of ER+ breast cancer cells mediated by interaction with macrophages. The cartoon depicts that the cytokines secreted by macrophages activates the TNF- and IL-6 receptors in the breast cells. This leads to the activation of NF-κB and STAT3 transcription factors together with the MAP kinases mediated phosphorylation of ERα at Ser-118. The combination of activated NF-κB, STAT3, and phospho-ERα is sufficient to induce the expression of key proliferative and proinflammatory genes in the breast cells.

Our findings clearly establish a role of conditioned macrophages in induction of endocrine resistance in ER+ breast cancer cells. Co-culture of macrophages with breast cancer cells induces sustained release of TNF-α and IL-6 from both cell types, resulting in activation of NF-κB, STAT3, and ERK-1 and hyperphosphorylation of ERα (rendering it constitutively active) in the breast cancer cells. Formation of an NF-κB/STAT3/phospho-ER complex in cyclin D1 gene was correlated with increased proliferation, independent of ER ligand status. A schematic model of macrophage effects on ERα+ breast cancer cells is shown in Figure 7.

A possible explanation of why ERα+ breast tumors stop responding to endocrine therapy is as follows. Estradiol suppresses NF-κB-responsive genes in breast cancer cells [61,97]. ER− breast cancer is associated with elevated NF-κB activity [32,63], and shows increased expression of certain cytokines (IL-6, IL-8) and chemokines (CCL5, MCP-1 [CCL2]) [64,65]. ER antagonists (e.g., tamoxifen, ICI 182,780) exert effects on breast cancer cells similar to those of aromatase inhibitors; i.e., they block the suppressive effect of estradiol on NF-κB-responsive genes [61,97]. These previous findings, in combination with ours, suggest that suppression of estrogen signaling by endocrine treatment leads to increased expression of NF-κB-responsive genes, promoting a proinflammatory microenvironment and infiltration of macrophages via expression of chemoattractants such as MCP-1. Macrophage infiltration is correlated with MCP-1 expression level, and both factors are clinically associated with worse prognosis in breast cancer [98,99]. Interaction between macrophages and ERα+ breast cancer cells in a proinflammatory microenvironment favors expression of TNF-α and IL-6. IL-6 and TNF-α then induce activation of STAT3 and NF-κB (respectively) via their receptors, and these receptors also activate ERK-1 cascade leading to ERα phosphorylation at Ser-118. NF-κB signaling pathway [31,32,63], IL-6/STAT3 signaling pathway [35], ER phosphorylation [25,26,27,28], and altered expression of cyclin D1 and c-Myc [35,36], have all been linked to endocrine resistance. Limited efficacy of tamoxifen treatment has been observed in clinical trials with numerous other types of ER+ cancer, including prostate, pancreatic, non-small cell lung, ovarian, and melanoma [100,101,102,103,104]. Improved long-term therapeutic efficacy will be achieved through combinations of classical chemotherapy and radiation therapy, anti-inflammatory therapy, and novel strategies targeting both tumor cells and their microenvironment. Interactions between tumor cells and their microenvironment, most of which involve macrophages, are highly complex. Elucidation of these complex interaction networks and their component signaling pathways, as in the present study, will lead to improved cancer therapeutic strategies in the future.

## 4. Materials and Methods

### 4.1. Cell Culture

Human breast cancer cell lines MCF-7, T47D, ZR75-1, BT474, SKBR3, MDA-231, HS578T, and HCC1395 were maintained in Dulbecco’s modified Eagle Medium (DMEM) without phenol red (Thermo Fisher, Waltham, MA, USA) plus 10% FBS (Gibco/Thermo Fisher), 1× MEM Non-Essential Amino Acids, and 1× PSN antibiotics (Thermo Fisher) at 37 °C in 5% CO_2_ atmosphere. Monocytic cell line THP-1 was maintained in RPMI 1640 medium (Gibco/Thermo Fisher) supplemented with 0.05 mM 2-mercaptoethanol, 1× PSN, and 10% FBS. Monocytic cell line KG-1 was maintained in DMEM supplemented with 1× PSN and 20% FBS. All cell lines were obtained from ATCC (Manassas, VA, USA) in 2015, cultured at 37 °C in 5% CO_2_ atmosphere until passage 2–4, and then cultured for 3–4 additional passages if necessary. Cells were authenticated on the basis of morphology and growth curve analysis. Mycoplasma detection was performed every 2 months by PCR and Hoechst 33,258 staining as described previously [105,106].

### 4.2. Primary Human Macrophage Culture and Differentiation

Monocytes were obtained from whole blood of anonymous healthy donors through the Blood Banking Facility, Hospital Nacional de Clínicas, Universidad Nacional de Córdoba (UNC). The protocol was approved by the Ethics Committee of Hospital Nacional de Clínicas (HNC) (GIL-PU-2015), UNC. Informed consent for all human-derived specimens was obtained from subjects in accordance with requirements of the Institutional Review Board of HNC, UNC. Low-density mononuclear cells were isolated from whole blood by standard gradient centrifugation with Ficoll-Paque Premium 1.073 g/mL (GE Healthcare Bio-Sciences, Pittsburgh, PA, USA). Monocytes were isolated by EasySep™ Human Monocyte Enrichment Kit (Stem Cell Technologies, Tukwila, WA, USA) as per the manufacturer’s protocol with minor modifications. Macrophages were obtained by differentiation of monocytes induced by hrM-CSF 10 ng/mL (Thermo Fisher) in 75% RPMI 1640 plus 15% Opti-MEM (Gibco) supplemented with 10% FBS and 1 × PSN. Adherent cells were collected after 3–5 days [107].

### 4.3. Differentiation and Conditioning of Macrophages

Primary monocytes were differentiated into macrophages using hrM-CSF 10 ng/mL as described above. Differentiation of monocyte lines KG-1 and THP-1 was induced by the method of Michiels’ group [108], i.e., treatment for 48 h with 10 ng/mL phorbol 12-myristate-13-acetate (PMA) followed by 24 h incubation in RPMI 1640 medium. Following differentiation, macrophages were treated with 1 ng/mL recombinant TNF-α (human) for 6 h at 37 °C in 5% CO_2_ atmosphere, to obtain conditioned macrophages, which were then washed and used in subsequent experiments as described below.

### 4.4. Macrophage-Conditioned Medium and Cell Coculture

Conditioned macrophages were cultured as described above. Culture medium was then collected, centrifuged at 300× g for 10 min at 4 °C to remove cell debris, and filtered twice (filter pore size 0.22 μm) to obtain macrophage-conditioned medium (CM), which was aliquoted and stored at −70 °C.

CM from macrophages co-cultured with MCF-7 cells was obtained by a similar procedure with some modification. Monocytes were differentiated into macrophages as previously, on top of a porous membrane (pore size 0.4 μm) in insertion well of 35-mm plate. This pore size allowed passage of chemical mediators and other solutes present in medium, but not of macrophages. Macrophages adhering to the membrane were conditioned with TNF-α as above, washed, and the insertion well was transferred to a different plate containing MCF-7 cells at 70–80% confluence. Macrophages and MCF-7 cells were cultured together in DMEM without phenol red, 1× MEM Non-Essential Amino Acids, and 1× PSN antibiotics for 24 h at 37 °C in 5% CO_2_ atmosphere. This CM from co-cultured macrophages was centrifuged, filtered, and stored at −70 °C.

### 4.5. Proliferation Assay

Breast cancer cells were seeded in 96-well plates (4000 cells/well). For co-culture with TNF-α conditioned macrophages, cells were placed in the upper well (~1000 cells/well) of a transwell Boyden chamber (pore size 0.4 µm; Corning Costar, Edison, NJ, USA). After 48 h, cell proliferation was assayed using CyQUANT NF Cell Proliferation Assay Kit (Thermo Fisher) as per the manufacturer’s protocol and results are expressed as arbitraries unit (a.u).

### 4.6. Colony Formation Assay

Breast cancer cells were cultured in the presence or absence of macrophages in MethoCult™ H4100 (Stem Cell Technologies) as per the manufacturer’s protocol with minor modifications. After 21 days, the colonies formed were counted under light microscopy.

### 4.7. Migration Assay

Migration was evaluated using 24-well plates and transwell Boyden chamber (Corning Costar) (pore size 8 μm) by the method of Maffucci’s group [109] with minor modifications. In brief, MCF-7 cells were pre-stained with CellTrace Calcein Red-Orange (AM) fluorophore (cat # C-34851, Thermo Fisher) by incubation with 1× fluorophore solution for 30 min at 37 °C in 5% CO_2_ atmosphere. Fluorescent cells were washed 3× with PBS, detached with 0.25% trypsin, and resuspended (density 1 × 10^6^ cells/mL) in DMEM without phenol red. One hundred μL of this suspension was placed on the membrane of the insertion well. The lower well was filled with culture medium containing various factors depending on the experiment, to a volume (~600 μL) allowing contact with the upper well. For macrophage co-culture experiments, KG-1 monocytes (4 × 10^4^) were differentiated with PMA in the lower well and conditioned with TNF-α for 6 h before and placing the insertion well. After 48 h culture, insertion wells were removed and fluorescence in the plate was quantified. Fluorescence intensity equal to that obtained by plating 1 × 10^5^ fluorescent cells directly in the lower well was defined as 100% migration.

### 4.8. Transfection of MCF-7 Cells

A gene encoding mutated IκBα (S32A and S36A), termed IκBα super-repressor, was cloned into vector pBabe-Puro-IKBalpha-mut in the EcoRI site. The plasmid (cat # 15291, Addgene, Watertown, MA, USA) was a gift from William Hahn. cDNAs of ERα refractory (ERαr) and mutated ERαr (S118A) were subcloned into pIRESneo-FLAG/HA EYFP vector at the EcoRI/BamHI site. The plasmid (cat # 10825, Addgene) was a gift from Thomas Tuschi. MCF-7 cells were transfected by standard protocol using transfection reagent FuGENE HD (cat # 04709705001, Roche, Pleasanton, CA, USA). In brief, 97 μL DMEM, 6 μL FuGENE reagent, and 1 μg plasmid DNA were mixed in that order in a sterile tube. The mixture was stirred vigorously for a few seconds, left for 20 min at room temperature, dripped onto MCF-7 cells (60–70% confluence) in a 35-mm plate, incubated for 8 h, and culture medium replaced by fresh medium.

### 4.9. siRNA

MCF-7 cells were transfected with ON-TARGETplus SMARTpool siRNAs (Dharmacon Inc, Lafayette, CO, USA), consisting of a mixed set of four siRNAs provided as individual reagents (ERα cat # 003401; STAT3 cat # 003544; GP-130 (IL6ST) cat # 005166), using XTREME (Roche) siRNA transfection reagent as per the manufacturer’s protocol with minor modifications. Following 72 h siRNA-mediated downregulation of gene expression, cells were treated as described in the figure legends.

### 4.10. Quantitative Real-Time RT-PCR (qPCR)

qPCR was performed using StepOnePlus Real Time PCR System (Thermo Fisher) and analyzed by StepOne software program, v. 2.1. Breast cancer cells (4000) were seeded in the bottom well of a 96-well transwell Boyden chamber, and macrophages were seeded in the upper well. Cells were treated as described in figure legends, and total mRNA was extracted after 48 h using RNeasy Micro Kit (Qiagen Inc, Valencia, CA, USA). One µg total mRNA was used for synthesis of cDNA by SuperScript III First-Strand Synthesis System (Thermo Fisher). ∆∆CT was obtained using 18S as endogen control. Gene expression-specific primers were obtained from TaqMan Gene Expression (Thermo Fisher) using NM_00000 gene IDs.

### 4.11. Chromatin Immunoprecipitation (ChIP) Assays

ChIP assays were performed using ChIP kit (cat # 17-295, MilliporeSigma, Burlington, MA, USA) as per the manufacturer’s protocol with minor modifications. All buffers used were from MilliporeSigma. For each eperimental condition, 3 × 10^6^ MCF-7 cells were synchronized by two days culture in DMEM/0.2% dextran-charcoal-treated FBS, then treated with 2.5 M amanitin for 2 h followed by treatment with 1 µM E2, 1 ng/mL TNF-α, 1 µM 4-OH-tamoxifen (Sigma-Aldrich, St. Louis, MO, USA), or various combinations as described in the figure legends. For macrophage co-culture experiments, KG-1 monocytes were cultured, differentiated, and TNF-α-conditioned in an insertion well (see above), treated with amanitin, and cultured together with MCF-7 cells. Cells were cross-linked (1.5% formaldehyde (Sigma-Aldrich), 10 min, room temperature), collected in collection buffer, incubated for 10 min on ice and then for 10 min at 30 °C, lysed by pipetting, centrifuged (2000× g) for 5 min at 4 °C with PBS, 1 mL lysis buffer A, and 1 mL buffer B, and sonicated 3×, 1 min each (Microson XL2000; 10–11 watts; setting # 4) in 500 µL lysis buffer. Fifty µL of each supernatant was used as input, and the remainder diluted 5-fold in immunoprecipitation (IP) buffer. This fraction was pre-cleared for 3 h at 4 °C with 2 g sheared salmon sperm DNA and 150 µL of 50% protein A-Sepharose bead (Pierce) slurry, and then subjected to IP overnight at 4 °C with rotary shaking. Antibodies were cross-linked to sepharose using Pierce IgG Plus Orientation kit (cat # 44990, Thermo Fisher), with polyclonal anti-rabbit IgG antibody (cat # ab171870, Abcam, Cambridge, MA, USA) as negative control. Complexes were recovered following 3 h incubation at 4 °C with 2 g salmon sperm DNA and 50 µL protein A-Sepharose bead slurry as above. Precipitates were washed serially with washing buffers I, II, and III (each 300 µL), then twice with 1 mM EDTA/10 mM Tris-HCl (pH 8.1). Precipitated complexes were separated from beads by three sequential incubations, each 10 min, with 50 µL of 1% SDS/0.1 M NaHCO3. Cross-linking was reversed by incubation overnight at 65 °C. DNA was purified using QIAquick columns (Qiagen). qPCR analysis was performed using 1 µL input material and 3 µL ChIP sample. qPCR primers were designed using File Builder software program v. 3.1 (Thermo Fisher). Response element binding sites were as described previously [32,61]. Values were calculated by fold-enrichment method relative to mock (IgG) using the formula 2^−(ΔCt),^ where ΔCt = Ctmock−Ctsample.

### 4.12. Antibodies Used for IP

Santa Cruz Biotechnology, Inc. Dallas, Texas, USA: ERα (HC-20) # sc-543, NF-κB p65 (C-20) # sc-372, NF-κB p65 (F-6) # sc-8008, STAT3 (C-20) # sc-482. Abcam, Cambridge, MA, USA: histone H3 (trimethyl K4) # ab8580, GFP antibody # ab290, NCoR # ab24552, KAT13A/SRC1 # ab2859. Thermo Fisher: CBP # PA5-27369. MilliporeSigma: RNA polymerase II (clone 8WG16) # 05-952, normal rabbit IgG # 12-370, normal mouse IgG # 12-371.

### 4.13. Cytokine, Growth Factor, and Chemokine Levels

Levels of these substances were assessed using immunoassay array (Human Cytokine Antibody Array 5, RayBio, Norcross, GA, USA) and quantified by ImageJ software program (https://imagej.nih.gov/ij/). Levels of individual cytokines were determined by quantitative ELISA (Thermo Fisher).

### 4.14. Animal Studies

Animal (mouse) studies and procedures were performed under supervision of the Dept. of Chemical Biology, Faculty of Chemical Sciences, UNC, in accordance with protocols approved by the Ethics Committee of the Dept. of Chemical Biology, and under protocol # 2141, approved by CICUAL (Comité Institucional para el Cuidado y Uso de Animales de Laboratorio) (www.fcq.unc.edu.ar/cicual). Mice were maintained under standard conditions. Athymic female Nu/Nu Nude (Crl:NU/NU-Foxn1nu) mice were housed under pathogen-free conditions and fed sterilized food and water. For xenograft experiments, animals were implanted subcutaneously with an estradiol pellet (0.72 mg/60 days slow release; cat # SE-121, Innovative Research). The next day, cancer cells were implanted alone, or mixed (ratio 1:4) with THP-1 cells in Matrigel (Becton Dickinson, Billerica, MA, USA) solution (1:1) and injected subcutaneously. Experimental treatments were initiated when tumor volume reached 500 mm^3^. Tumors were measured every three days using precision calipers, and their volume was calculated as $\frac{\pi }{6}*L*{\left(S\right)}^{2}$ mm^3^ (L = larger diameter; S = smaller diameter).

### 4.15. Western Blotting Analysis

Western blotting was performed by standard procedures. Primary antibodies used were directed to ERα (cat # 2512, Cell Signaling Technology, Danvers, MA, USA), phospho-S118 ERα (cat # ab32396, Abcam, Cambridge, MA, USA), and NF-κB p65 (cat # 4764, CST). Secondary antibodies were goat anti-rabbit and anti-mouse IgG conjugated to biotin (cat #s BA-1000, BA-9200, Vector Laboratories Inc, Burlingame, CA, USA) and or to HRP-streptavidin (cat # RPN1231, GE Healthcare Bio-Sciences, Pittsburgh, PA, USA). SuperBlock T20 PBS Blocking Buffer and antibody diluents (cat #37516, Thermo Fisher) were used, and blots were developed with ECL Plus (GE Healthcare Bio-Sciences).

### 4.16. Tumor Tissues, Immunohistochemistry, and Immunofluorescence

Tumor tissue samples were collected surgically and processed/evaluated by an experienced pathologist. Samples were fixed in 10% neutral buffered formalin for standard histological analysis, immunohistochemistry (IHC), and immunofluorescence (IF). Sections of paraffin-embedded tissue blocks (4 μm thick) were deparaffinized in xylene and rehydrated through graded alcohol series. For antigen retrieval, sections were immersed in 10 mM sodium citrate buffer (pH 6) and microwaved (750 W) for 15 min. Slides were blocked for 30 min in Modified Hanks’ Buffer with 5% BSA. For IHC, endogenous peroxidase activity was inhibited by 5% H_2_O_2_, and a blocking step with biotin-containing BSA was performed prior to primary antibody step. Primary anti-cytokeratin 7 (CK7) (OV-TL 12/30, mouse monoclonal; Cell Marque/Sigma-Aldrich) was diluted 1:100, incubated 1 h at room temperature, and detected using secondary biotinylated antibody and streptavidin/peroxidase conjugate system, with hematoxylin as nuclear counterstain. Negative controls were performed without primary antibodies, using 3,3′-diaminobenzidine as chromogen.

For IF, slides were incubated overnight at 4 °C with anti-human mouse mAb CD68 (clone KP-1, Thermo Fisher), diluted 1:100, as primary antibody. Slides were washed 3x with PBS and incubated for 1 h at room temperature with secondary antibody conjugated to Alexa 546 fluorochrome (Thermo Fisher), diluted 1:1000. Nuclei were stained with 4′,6-diamidino-2-phenylindole (DAPI; Sigma-Aldrich), and sections were counterstained with hematoxylin/eosin. Slides were examined under laser microscopy (Olympus), and immunostaining results were assessed in blinded manner by the pathologist.

### 4.17. Flow Cytometry

THP-1 macrophages were subjected to various treatments as described in figure legends, incubated with StemPro Accutase (Thermo Fisher) for 20 min at room temperature, and then incubated for 30 min at 4 °C with APC-labeled anti-CD86 antibody (cat # 305412, BioLegend, San Diego, CA, USA), or APC-Cy7-labeled anti-CD206 antibody (cat # 321120, BioLegend) as per the manufacturer’s protocol. Cells were washed, acquired on FACS Canto II (BD Bioscience, San Jose, CA, USA), and analyzed using FlowJo software program (Tree Star; Ashland, OR, USA).

### 4.18. Statistical Analysis

Experimental values are presented as mean ± standard error of the mean (SEM). At least three independent trials were performed for each experiment. Statistical analytical Methods and numbers of data points analyzed for each experiment are described in figure legends. Statistically significant analysis by two-way ANOVA with Sidak multiple comparison test (α = 0.05) was performed in experiments graphed in Figure 1a–d, Figure 2a, Figure 3a–f, Figure 4a,e, Figure 5a–e and Figure 6a–c,e–j. One-way ANOVA with Dunnett’s test (α = 0.05) was performed in experiments graphed in Figure 1e, Figure 2b and Figure 6d. For qPCR results, only log2 fold changes ≤ −0.15 or ≥ 0.15 were tested for statistical significance. Graphs were generated using GraphPad Prism for Mac OS X, v. 7.0d (GraphPad Software; La Jolla, CA, USA). For all analyses, differences with *p* ≤ 0.05 were considered significant.

## 5. Conclusions

Crosstalk between conditioned macrophages and ER+ breast cancer cells in vitro promotes formation of a microenvironment in which proliferation, migration, and invasion of tumor cells are enhanced even in the presence of ER antagonists. Endocrine resistance to tamoxifen or ICI 182,780 treatment develops even without direct contact between cancer cells and macrophages, indicating that the process is based on chemical mediators released into culture medium. In this study, co-culture of conditioned macrophages and breast cancer cells resulted in strong, sustained increase of TNF-α and IL-6 protein levels. TNF-α/NF-κB and IL-6/STAT3 pathways played essential roles in macrophage-mediated endocrine resistance. Binding of transcription factors NF-κB and STAT3 to promoters of key proliferative genes such as cyclin D1 and c-Myc, in combination with ERα, provided the basis for estradiol-independent, non-canonical proliferation of ER+ breast cancer cells. The microenvironment generated by interactions between macrophages and ER+ breast cancer cells, both in vitro and in vivo, controls the key signaling pathways that induce endocrine resistance even in the absence of estrogen.

## Supplementary Materials

The following are available online at http://www.mdpi.com/2072-6694/11/2/189/s1, Figure S1: Macrophages-mediated endocrine resistance in different breast cancer cells, Figure S2: Requirement for c-Myc and cyclin D1 for breast cancer cell proliferation, Figure S3: ChiP analyses of c-Myc promoter, Figure S4: Culture medium-Cytokine analysis in MCF-7, KG-1 macrophages or co-culture of both cell lines, Figure S5: STAT3 down regulation in MCF-7 does not directly affect the level of ER expression and vice versa, Figure S6: Analysis of the profile of THP-1 macrophages co-cultured with MCF-7 cells, Figure S7: KG-1 macrophages increase the interaction between p65, STAT3, and ERα proteins in MCF-7.

## Abbreviations

a.u = arbitraries unit; CBP = CREB-binding protein; CCL2 = chemokine (C-C motif) ligand 2; a.k.a. MCP-1 (monocyte chemoattractant protein 1); CCL5 = chemokine (C-C motif) ligand 5 (a.k.a. RANTES); ChIP = chromatin immunoprecipitation; CK7 = Primary anti-cytokeratin 7; CM: conditionate media; DMEM = Dulbecco’s modified Eagle medium; DNA = deoxyribonucleic acid; E2 = estradiol; EDTA = ethylenediaminetetraacetic acid; ELISA = enzyme-linked immunosorbent assay; ERK-1 = extracellular signal-regulated kinase-1; ERα = estrogen receptor alpha; hrM-CSF = human recombinant-macrophage colony-stimulating factor; ICI 182,780 (a.k.a. Faslodex, Fulvestrant) = estrogen receptor antagonist; IkBα = nuclear factor of kappa light polypeptide gene enhancer in B-cells inhibitor, alpha; IL-1α = interleukin 1 alpha (a.k.a. hematopoietin 1); IL-1β = interleukin 1 beta (a.k.a. leukocytic pyrogen, or leukocytic endogenous mediator); IL-6 = interleukin 6; IL-8 = interleukin 8 (a.k.a. CXCL8 [chemokine (C-X-C motif) ligand 8]); NF-κB = nuclear factor kappa-light-chain-enhancer of activated B cells; NT-3 = neurotrophin-3; qPCR = quantitative real-time reverse transcription polymerase chain reaction; SDS = sodium dodecyl sulfate; Ser = serine; SERMs = selective estrogen receptor modulators; siRNA = small interfering RNA; SRC1 = steroid receptor coactivator-1 (a.k.a. NCOA1 [nuclear receptor coactivator 1]); STAT3 = signal transducer and activator of transcription 3; TNF-α = tumor necrosis factor alpha; TPA = 12-O-tetradecanoylphorbol-13-acetate.
