# Supplementary material for: Tumor-Associated Macrophages Induce Endocrine Therapy Resistance in ER+ Breast Cancer Cells
Source: Cancers (Basel). 2019 Feb 6;11(2):189. doi: 10.3390/cancers11020189 (PMC6406935; doi:10.3390/cancers11020189)
Supplement: Supplementary file 1 [file cancers-11-00189-s001.pdf]

Supplementary Materials

Tumor-Associated Macrophages Induce Endocrine Therapy Resistance in ER+ Breast Cancer Cells

Andrés M. Castellaro, María C. Rodriguez-Baili, Cecilia E. Di Tada and Germán A. Gil

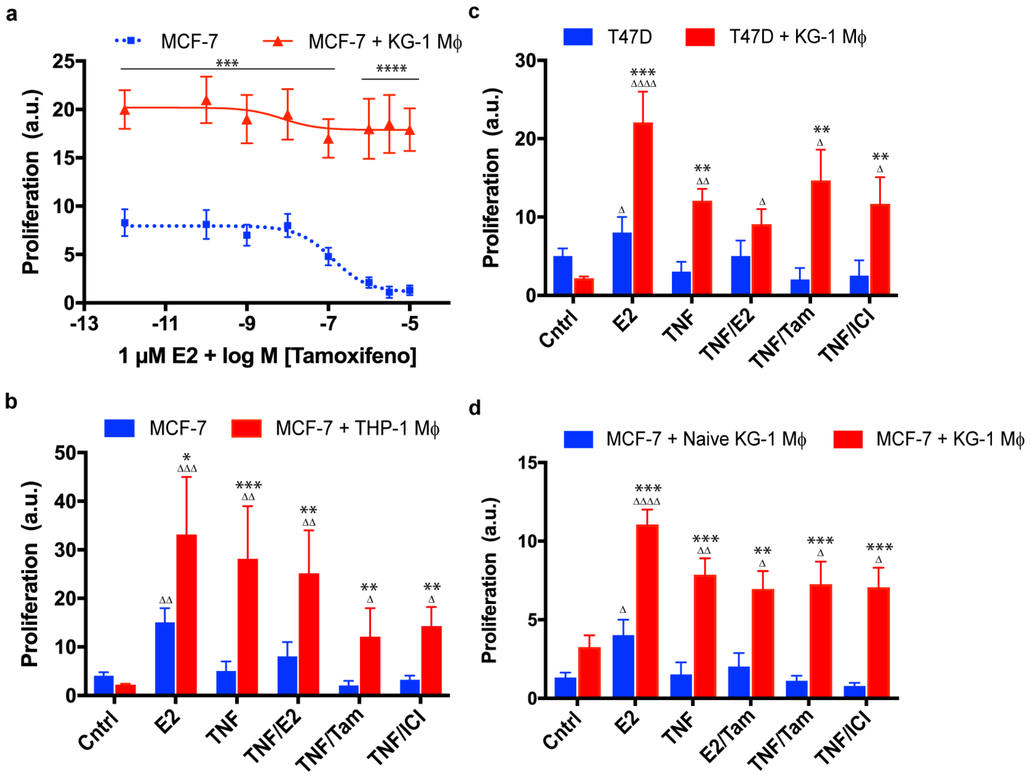

Figure S1. Macrophages-mediated endocrine resistance in different breast cancer cells. (**a**) Proliferation of MCF-7 cultured in the presence or the absence of conditioned KG-1 macrophages. Cell cultures were separated by a semipermeable membrane and cultured for two days stimulated with E2 (1 µM) and treated with an increasing concentration of tamoxifen as indicated in the figure and measured by CyQUANT. $n = 3$. Analysis was performed by two-way ANOVA with Sidak multiple comparison test ($\alpha = 0.05$) and compared with either, MCF-7 or macrophages; (**b**) Proliferation of MCF-7 cultured in the presence or the absence of conditioned THP-1 macrophages and measured by CyQUANT. Cell cultures were separated by a semipermeable membrane and cultured for two days with the indicated ligands, $n = 3$. Analysis as in panel **a**: * in comparison with absence of macrophages in indicated treatment; Δ in comparison with the respective Cntrl of each group; (**c**) Proliferation of T47D cells cultured in presence or absence of conditioned KG-1 macrophages was measured in the same conditions as **b**. Analysis was performed as in panel **a**: * in comparison with absence of macrophages in indicated treatment; Δ in comparison with respective Cntrl of each group; (**d**) Proliferation of MCF-7 plus naïve THP-1 macrophages or MCF-7 plus conditioned THP-1 macrophages was measured in the same conditions as **b**. Analysis as in panel **a**: * in comparison with absence of macrophages in indicated treatment; Δ in comparison with respective Cntrl of each group. References: Cntrl: Fresh DMEM, E2: Estradiol 1 nM, TNF: TNF-α 1 ng/mL, Tam: Tamoxifen 1 µM, ICI: ICI 182,780 1 µM. Shown are the mean ± SEM from 3 independent experiments. * $p < 0.05$; ** $p < 0.01$; *** $p < 0.001$; **** $p < 0.0001$; Δ $p < 0.05$; ΔΔ $p < 0.01$; ΔΔΔ $p < 0.001$; ΔΔΔΔ $p < 0.0001$.

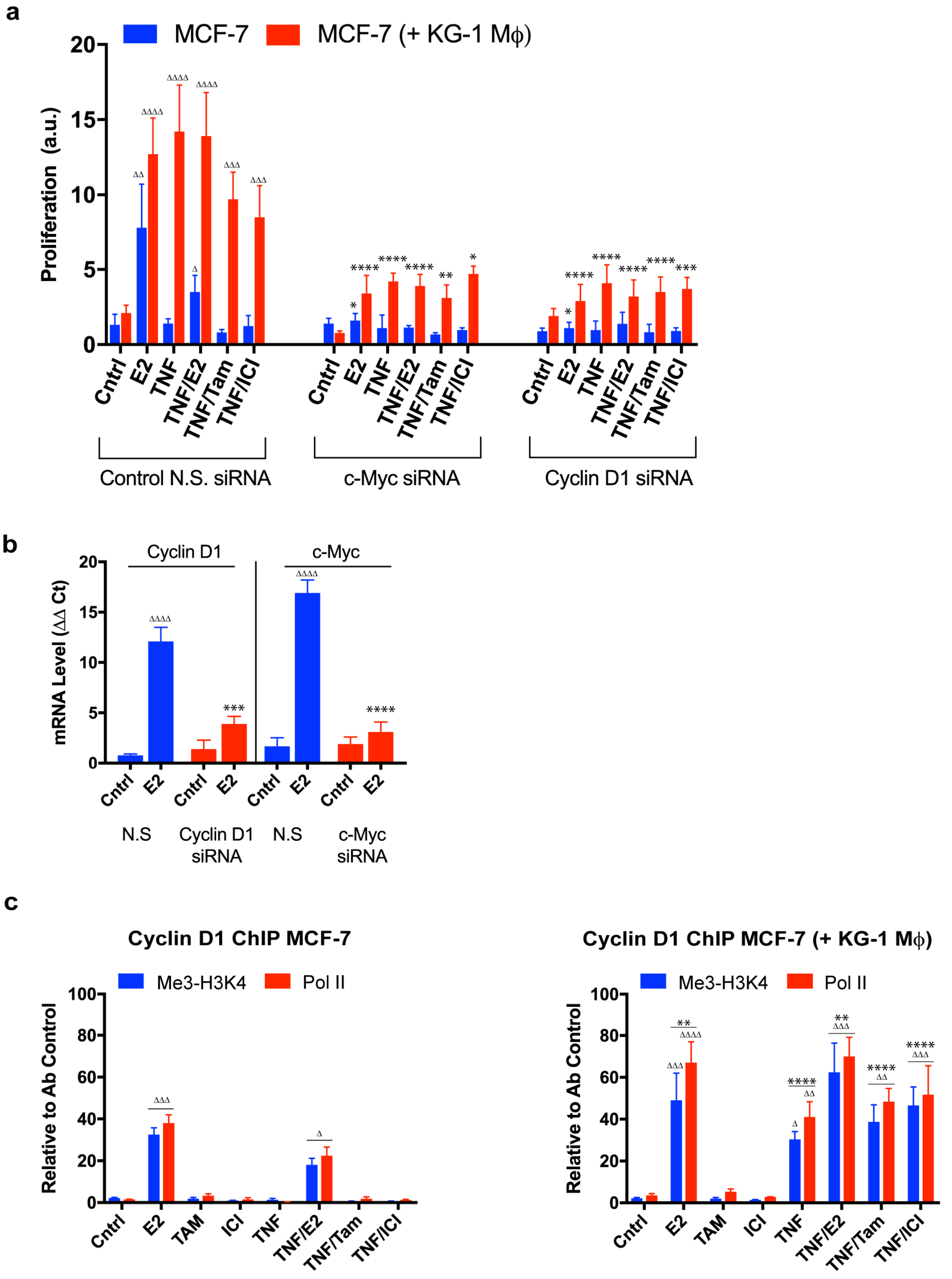

Figure S2. Requirement for c-Myc and cyclin D1 for breast cancer cell proliferation. (**a**) Proliferation of MCF-7 transfected with a siRNA targeting c-Myc, cyclin D1, or with N.S. siRNA as control and incubated in presence or absence of conditioned KG-1 macrophages for 48 h with the indicated ligands and measured by CyQUANT, $n = 3$. Analysis was performed by two-way ANOVA with Sidak multiple comparison test ($\alpha = 0.05$): * in comparison with Control N.S. siRNA in indicated treatment of each group; e.g., TNF siRNA c-Myc vs. TNF Control N.S. siRNA; Δ in comparison with respective Cntrl of each group; (**b**) Cyclin D1 or c-Myc gene expression in MCF-7 transfected with siRNA targeting cyclin D1, c-Myc or N.S. siRNA. MCF-7 were cultured in the presence of conditioned KG-1 macrophages for 48 h. Cells were treated as indicated for 2 h prior to be harvested and processed for qPCR. Relative mRNA expression with respect to control (N.S. siRNA) was calculated by the $2^{-\Delta\Delta Ct}$ method $n = 4$. Analysis as in panel **a**: Δ in comparison with Cntrl of each group; * in comparison with N.S.siRNA in indicated treatment of cyclin D1 or c-Myc; (**c**) ChIP assay of trimethylated Lys-4 on Histone-3 (Me3-H3K4) and RNA Polymerase II (Pol II) followed by qPCR analysis of the cyclin D1

promoter in MCF-7 cultured alone (left) or in the presence of conditioned KG-1 macrophages (right) for 24 h. The cultures received the indicated treatments 2 h prior to harvesting. Results are normalized to non-specific IgG Ab, $n = 4$. Analysis as in panel a: Δ in comparison with Cntrl of each group; * in comparison with absence of macrophages of each group; e.g., E2 (Pol II, MCF-7 + Macrophages) vs. E2 (Pol II, MCF-7). References: Cntrl: Fresh DMEM, E2: Estradiol 1 nM, TNF: TNF-α 1 ng/mL, Tam: Tamoxifen 1 µM, ICI: ICI 182,780 1 µM. Shown are the mean ± SEM from 3 independent experiments. * $p < 0.05$; ** $p < 0.01$; *** $p < 0.001$; **** $p < 0.0001$; Δ $p < 0.05$; ΔΔ $p < 0.01$; ΔΔΔ $p < 0.001$; ΔΔΔΔ $p < 0.0001$.

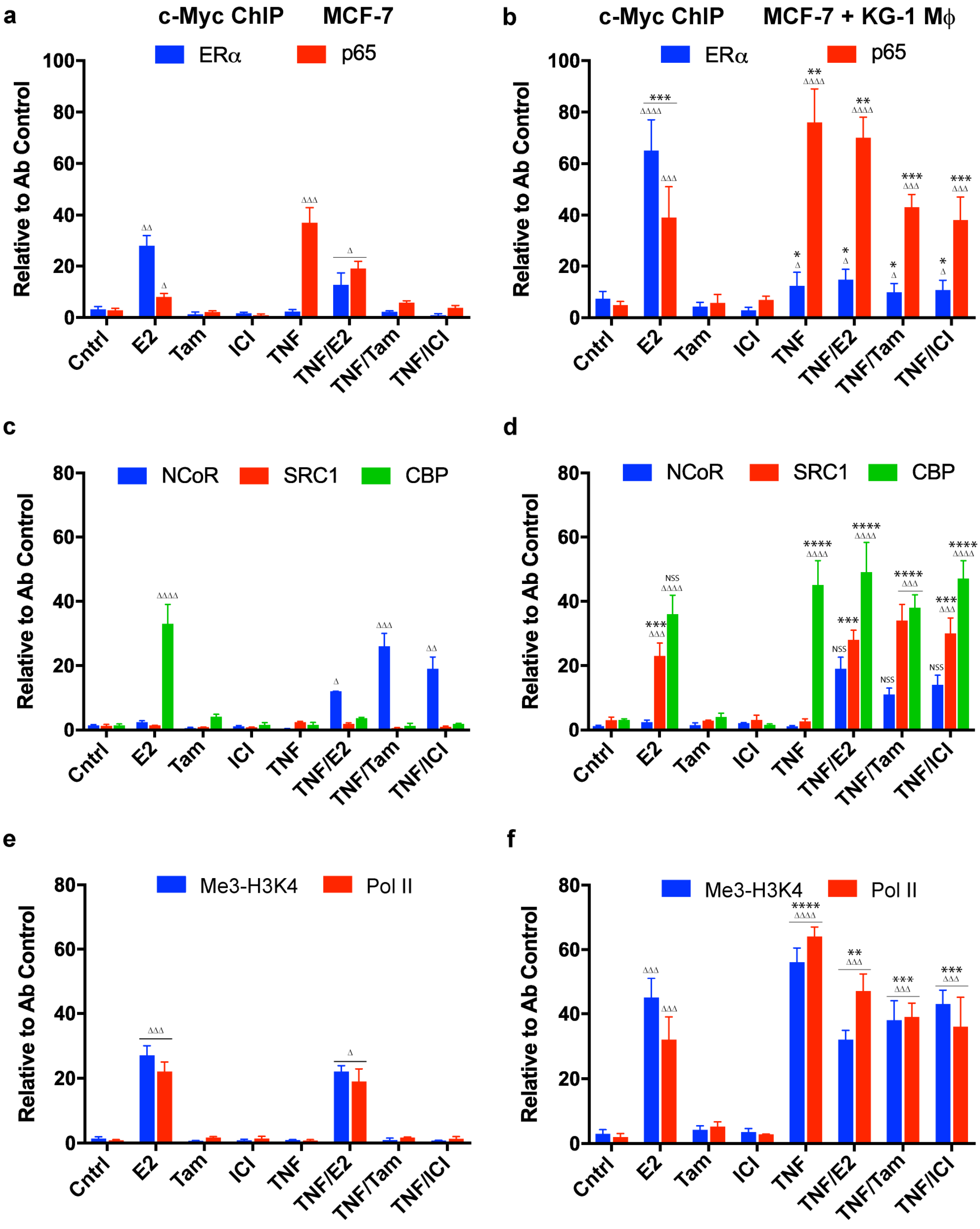

Figure S3. ChiP analyses of c-Myc promoter. ChiP assay of (**a**,**b**) ERα, and p65; (**c**,**d**) NCOR, SRC1, and CBP; (**e**,**f**) trimethylated Lys-4 on Histone-3 (Me3-H3K4), and RNA Polymerase II (Pol II) followed by qPCR of the cyclin D1 promoter in MCF-7 cultured alone (**a**,**c**,**e**) or in the presence of conditioned KG-1 macrophages (**b**,**d**,**f**) for 24 h. The cultures received the indicated treatments 2 h prior to harvesting. Results are normalized to non-specific IgG Ab, $n = 4$. Analysis was performed by

two-way ANOVA with Sidak multiple comparison test ($\alpha = 0.05$): Δ in comparison with Cntrl of each group; * in comparison with absence of macrophages in indicated treatment of each group; e.g., E2 (ERα MCF-7+ Macrophages) vs. E2 (ERα MCF-7). References: Cntrl: Fresh DMEM, E2: Estradiol 1 nM, TNF: TNF-α 1 ng/mL, Tam: Tamoxifen 1 μM, ICI: ICI 182,780 1 μM. Results are the mean ± SEM of three experiments performed. * $p < 0.05$; ** $p < 0.01$; *** $p < 0.001$; **** $p < 0.0001$; Δ $p < 0.05$; ΔΔ $p < 0.01$; ΔΔΔ $p < 0.001$; ΔΔΔΔ $p < 0.0001$; NSS: not statistically significant.

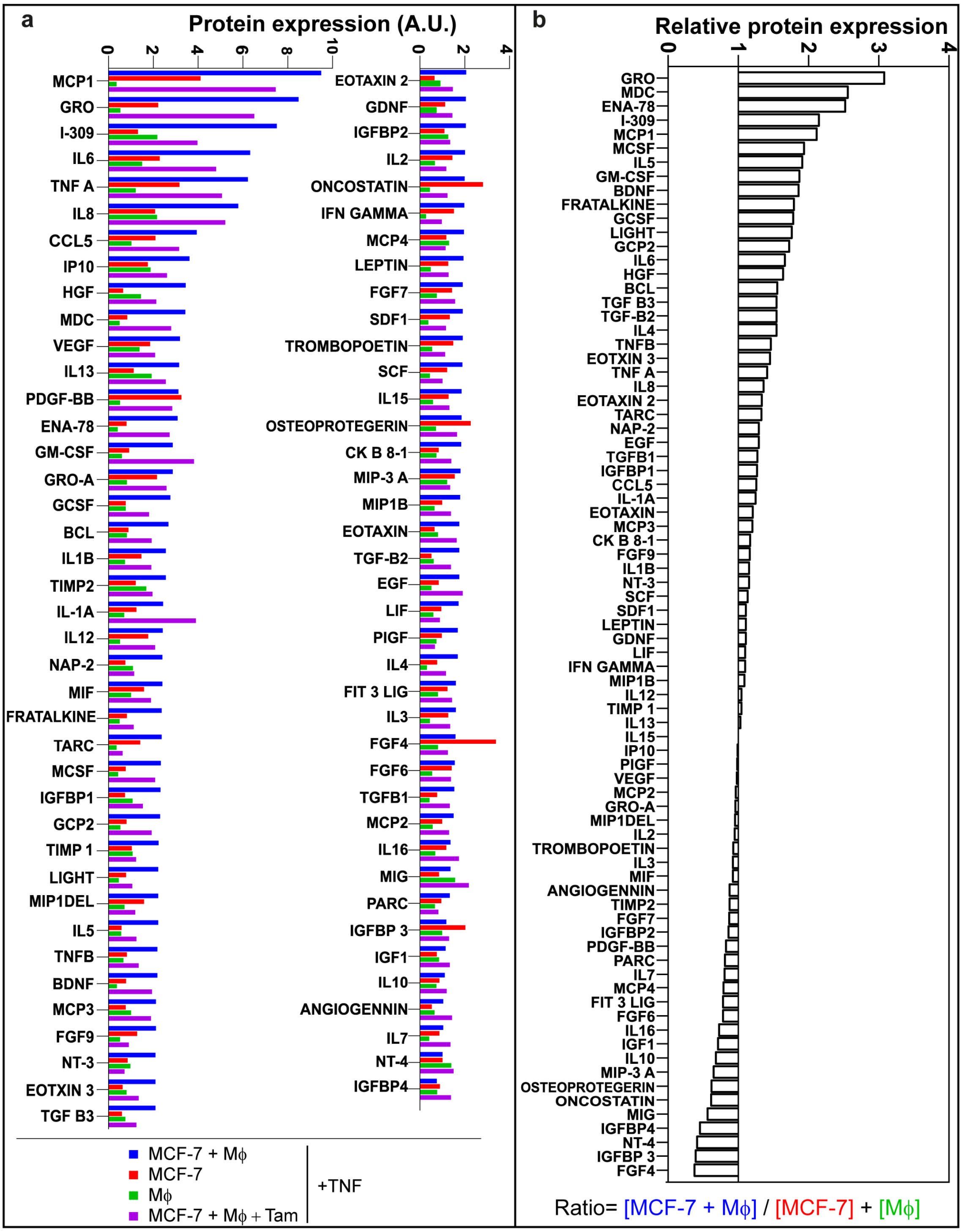

Figure S4. Culture medium-Cytokine analysis in MCF-7, KG-1 macrophages or co-culture of both cell lines. MCF-7, KG-1 macrophages and MCF-7-KG-1 macrophages co-cultured were treated with TNF (1 ng/mL) for 6 h and then washed. The media was collected 24 h later and analyzed for cytokine antibody protein array. Unstimulated cells were used as control. (**a**) Shown are results with over two-

fold TNF-α induction relative to control. (**b**) Shown is the ratio of each cytokine in the co-culture with respect to the sum of the individual cultures; e.g., ratio = [(MCF-7 + Mϕ) /(MCF-7) plus (Mϕ)].

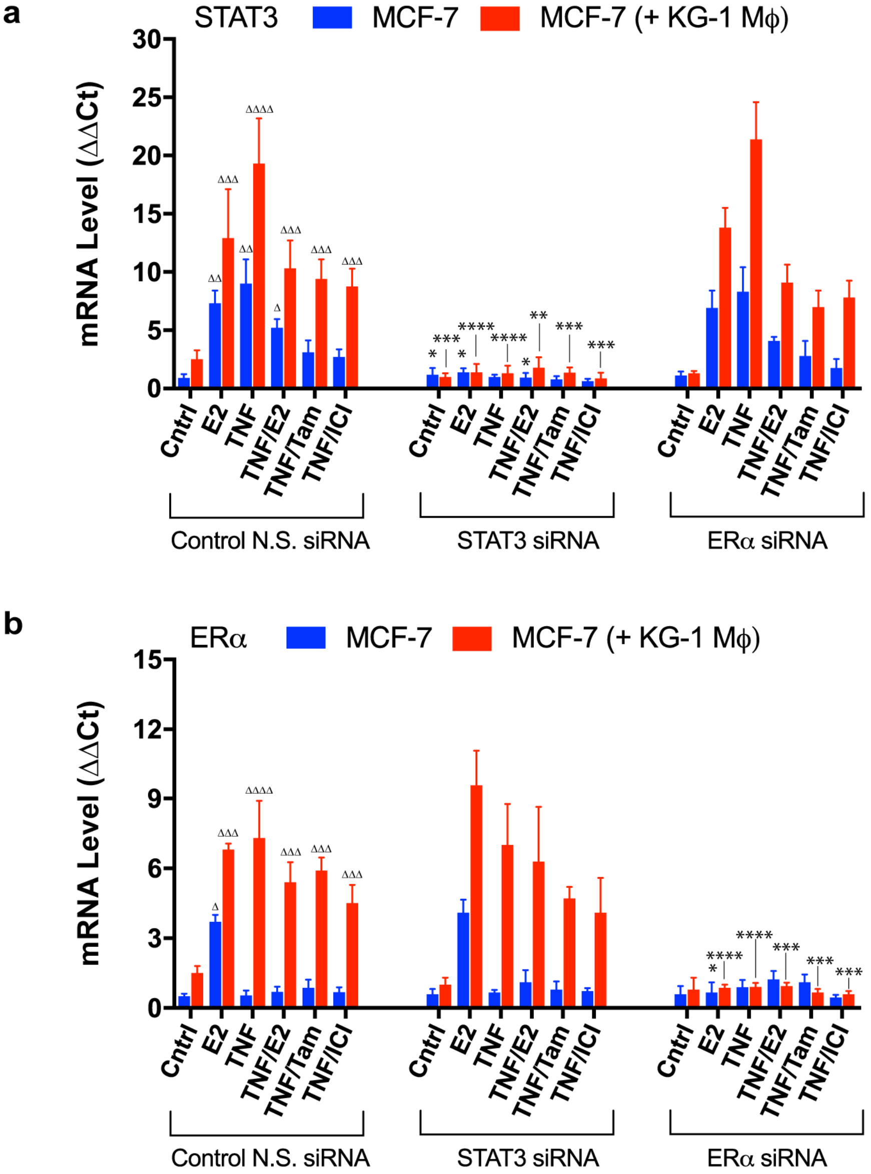

Figure S5. STAT3 down regulation in MCF-7 does not directly affect the level of ER expression and vice versa. (**a**,**b**) Expression level of the Stat3 (**a**) and ERα (**b**) genes in MCF-7, following 2 h of treatment with the indicated ligands. MCF-7 were previously transfected with siRNA Stat 3, ERα or N.S. siRNA and cultured in presence or absence of KG-1 macrophages by two days. Relative mRNA expression with respect to control (N.S. siRNA) was calculated by the $2^{-\Delta\Delta Ct}$ method $n = 4$. Analysis by two-way ANOVA with Sidak multiple comparison test ($\alpha = 0.05$): * in comparison with control N.S. siRNA in indicated treatment; **Δ** in comparison with respective Cntrl of each group. References: Cntrl: Fresh DMEM, E2: Estradiol 1 nM, TNF: TNF-α 1 ng/mL, Tam: Tamoxifen 1 µM, ICI: ICI 182,780 1 µM. Results are the mean ± SEM of three experiments performed. * $p < 0.05$; ** $p < 0.01$; *** $p <$ 0. 001; **** $p <$ 0. 0001; Δ $p < 0.05$; ΔΔ $p < 0.01$; ΔΔΔ $p < 0.001$; ΔΔΔΔ $p < 0.0001$.

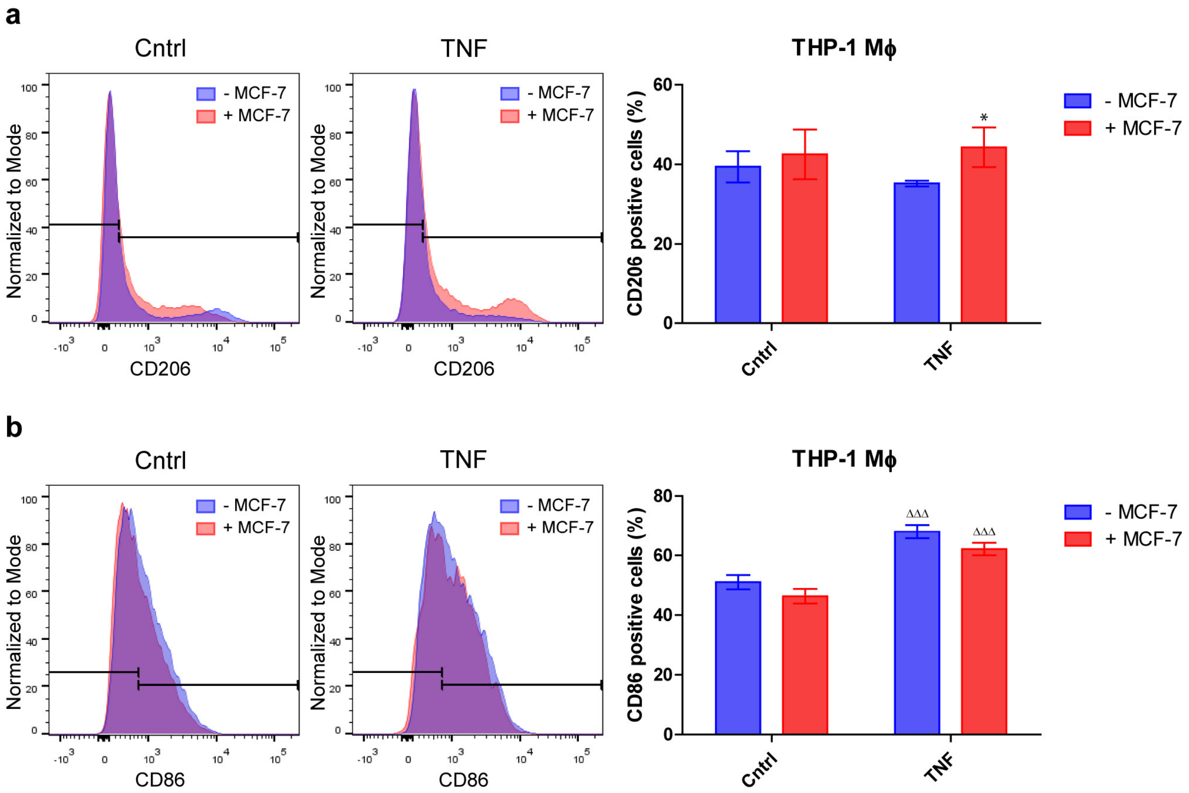

Figure S6. Analysis of the profile of THP-1 macrophages co-cultured with MCF-7 cells. (**a**,**b**) The expression level of CD206 (**a**) and CD86 (**b**) proteins was assessed in THP-1 macrophages cultured for 48 h in presence or absence of MCF-7 separated by a semipermeable membrane (pore size 0.4 µm). Cells were pretreated or not with TNF for 6 h before been co-cultured. The bar graphs show the percentage of the population of THP-1 macrophages positive for CD206 or CD86 measured by flow cytometry, $n = 6$. Analysis by two-way ANOVA with Bonferroni multiple comparison test ($\alpha = 0.05$): Δ in comparison with Cntrl of each group; * in comparison with absence of macrophages in indicated treatment. References: Cntrl: Fresh DMEM, TNF: TNF-α 1 ng/mL. Results are the mean ± SEM of three experiments performed. * $p < 0.05$; ΔΔΔ $p < 0.001$.

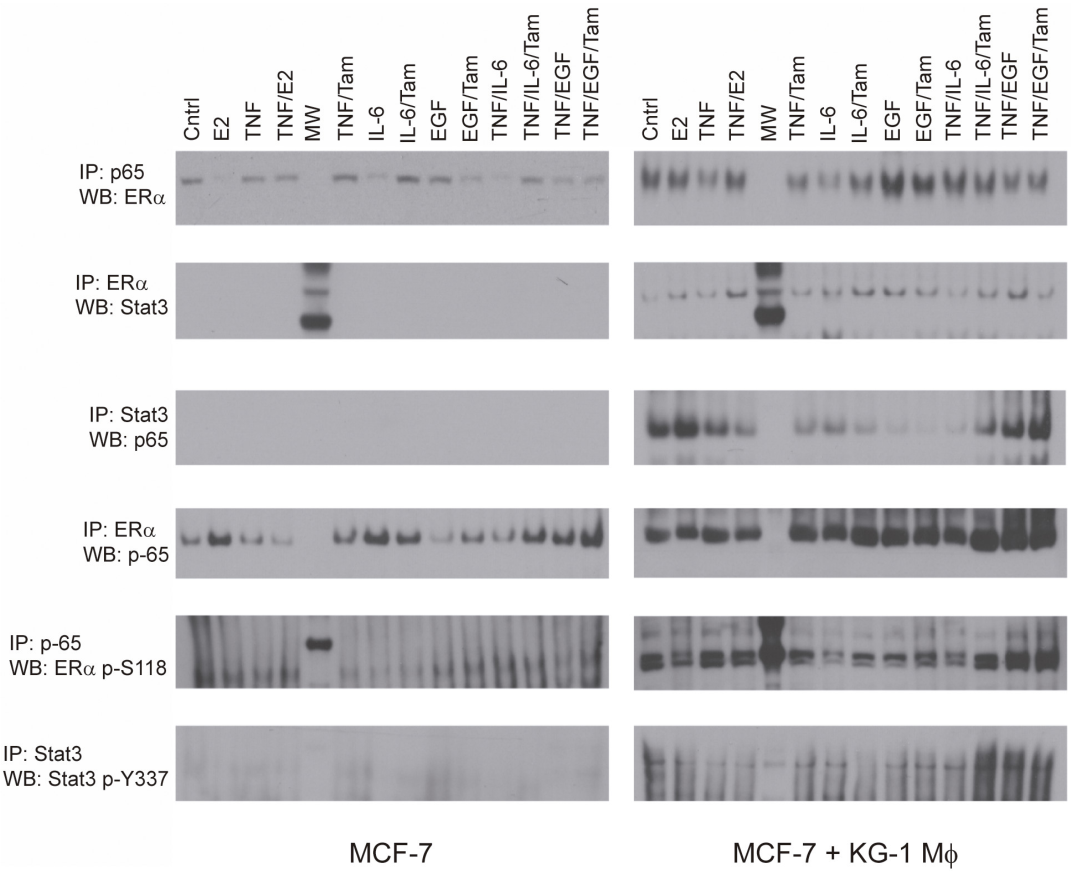

Figure S7. KG-1 macrophages increase the interaction between p65, STAT3, and ERα proteins in MCF-7. IP of p65, STAT3, or ERα followed by Western blotting (WB) of MCF-7 cultured in the presence or the absence of conditioned KG-1 macrophages separated by a semipermeable membrane for 24 h. Cells were treated 2 h before harvesting as indicated in each lane. References: Cntrl: Fresh DMEM, E2: Estradiol 1 nM, TNF: TNF-α 1 ng/mL, IL-6: Interleukin 6 1 ng/mL, EGF: Epidermal growth factor 10 ng/mL, Tam: Tamoxifen 1 μM, MW: Molecular weight marker.
